# Open source high-temperature RepRap for 3-D printing heat-sterilizable PPE and other applications

**DOI:** 10.1016/j.ohx.2020.e00130

**Published:** 2020-07-30

**Authors:** Noah G. Skrzypczak, Nagendra G. Tanikella, Joshua M. Pearce

**Affiliations:** a Mechanical Engineering – Engineering Mechanics, Michigan Technological University, USA; b Department of Materials Science & Engineering, Michigan Technological University, USA; c Department of Electrical & Computer Engineering, Michigan Technological University, USA; d Équipe de Recherche sur les Processus Innovatifs (ERPI), Université de Lorraine, France; e School of Electrical Engineering, Aalto University, Finland

**Keywords:** Open source, Open hardware, COVID-19, Medical hardware, RepRap, 3-D printing, Open source medical hardware, High temperature 3-D printing, Additive manufacturing, ULTEM, Polycarbonate

## Abstract

Thermal sterilization is generally avoided for 3-D printed components because of the relatively low deformation temperatures for common thermoplastics used for material extrusion-based additive manufacturing. 3-D printing materials required for high-temperature heat sterilizable components for COVID-19 and other applications demands 3-D printers with heated beds, hot ends that can reach higher temperatures than polytetrafluoroethylene (PTFE) hot ends and heated chambers to avoid part warping and delamination. There are several high temperature printers on the market, but their high costs make them inaccessible for full home-based distributed manufacturing required during pandemic lockdowns. To allow for all these requirements to be met for under $1000, the Cerberus – an open source three-headed self-replicating rapid prototyper (RepRap) was designed and tested with the following capabilities: i) 200 °C-capable heated bed, ii) 500 °C-capable hot end, iii) isolated heated chamber with 1 kW space heater core and iv) mains voltage chamber and bed heating for rapid start. The Cereberus successfully prints polyetherketoneketone (PEKK) and polyetherimide (PEI, ULTEM) with tensile strengths of 77.5 and 80.5 MPa, respectively. As a case study, open source face masks were 3-D printed in PEKK and shown not to warp upon widely home-accessible oven-based sterilization.

## Graphical abstract

**Figure:**
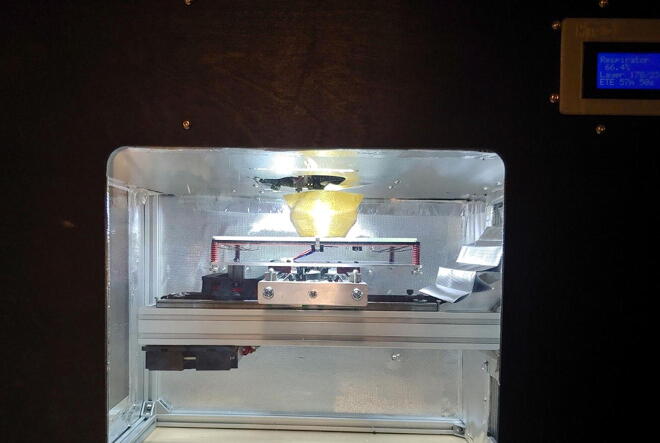

Specifications

**Table:** 

Hardware name	Cerberus - Open Source High Temperature 3-D Printer
Subject area	• Engineering and Material Science
Hardware type	• Additive manufacturing
Open Source License	GNU General Public License (GPL) v3.0 and CERN Open Hardware License (OHL) v1.2
Cost of Hardware	Under $1000
Source File Repository	Registration: https://osf.io/46njf
	Repository: https://osf.io/gbjvf/

## 1 Hardware in context

Coronavirus disease 2019 (COVID-19) caused by the SARS-CoV-2 virus [1] has overwhelmed medical infrastructure at the regional level [2] because of shortages [3]. The rapid spread of COVID-19 created temporary shortages of medical equipment like ventilators in the U.S. [4] and in Europe [5] and personal protective equipment (PPE) locally [6], [7] as well as globally as reported by the World Health Organization [8]. There has been an explosion of distributed manufacturing [9] (where products are made at the local level) by small companies [10], conventional makerspaces and fab labs [11] green fab labs [12]. Distributed manufacturing can even occur at the personal level using desktop manufacturing [13] with substantial consumer savings for a wide range of products [14], [15] including personal equipment [16] and flexible products [17]. Distributed manufacturing can also be used to overcome these shortages of medical supplies in general [18] and specifically for medical face shields [19], [20] (about 60% of total [21]), respirator materials [22], and ventilators [23], [24]. Distributed manufacturing with 3-D printing has been used to effectively manufacture custom parts [25] for breathing apparatuses [26] and perhaps most importantly for PPE to help reduce the spread of the disease including facemasks [27], face shields [19], [20], [21], [28], helmet modifications [29], door openers [30] and reusable N95 respirators [31].

The vast majority of this 3-D printed PPE is meant to be disposable after a single use (e.g. shields and facemasks) and there are substantial challenges to reprocessing and reusing 3-D printed PPE [32]. One approach investigated the use of antimicrobial polymers [32] but has not been shown to be effective against viruses. Levingston, Desai and Berkwits [6] report that although there have been numerous suggestions to sterilize PPE including ethylene oxide, hydrogen peroxide and UV [33] or gamma irradiation, ozone, and alcohol prior, previous work from other viral epidemics provides some guidance [34], but overall there is uncertainty. Rubio-Romero et al., report that the best methods for sterilizing normally-disposable face masks is methods are those that use hydrogen peroxide vapor, ultraviolet radiation, moist heat, dry heat and ozone gas [35]. 3-D printing helps extend supply of one-time-use PPE, and reusable face-masks that need only a fraction of the filter material of an N95 mask have the potential to greatly expand supply [36]. This method, however is challenging to implement because of the wide variation in 3-D printer/user capability and thus resultant part quality but also because of the porous nature of material extrusion-based 3-D printing (which can even be influenced by the color of the filament [37]) make some of these techniques questionable [38]. For some PPE like widely 3-D printed face shields [39], [40] chemical methods may be adequate, but that for masks reasonably straight-forward methods like soapy water, alcohol, bleach immersion, ethylene oxide, and ionizing radiation are not fully recommended [35]. Instead for face masks even if virus particles made their way inside porous media, using hot air is considered the most effective method for home disinfection [35]. **A low-cost 3-D printer is needed that can print high-temperature polymers that would enable fabrication of heat sterilizable PPE.**

Sterilization using high temperatures is not viable for common methods of 3-D printing, which are generally material extrusion fused filament fabrication (FFF)/fused deposition modeling (FDM) because of the relatively low-melting points (and deformation temperatures) of commonly used plastics. Normally FFF-based printers print thermopolymers like poly lactic acid (PLA), acrylonitrile butadiene styrene (ABS), and glycol modified version of polyethylene terephthalate (PETG), the latter of which has emerged as the printing material of choice for most COVID-19 projects. Although there are other 3-D printing methods, FFF is the most widely accessible additive manufacturing technology because of the democratization and resultant low-cost evolution of the open source release of the self-replicating rapid prototyper (RepRap) project [41], [42], [43]. Open source hardware design [44] and distributed reproduction with RepRaps and their derivatives are used in medical applications [45], [46] have been applied to on sight medical equipment [47] with good economics [48]. This approach is adept at overcoming supply shortages for humanitarian logistics [49], [50], disaster preparedness [51], humanitarian response [52] and for rural health care [53]. This previous work, however, has focused on low-temperature melting plastics (e.g. PLA, ABS PETG, and other common commercial filaments like nylons and thermoplastic urethane (TPU)). This is again, because of the generally high-costs and low accessibility of 3-D printers rated for high temperature (i.e. Aniwaa, for example, lists 7 high-temperature 3-D printers commercially available in 2020 with prices that range from $25,000 to $110,000 with most costing over $50,000 [54]). High-costs of such high-temperature printers exists because of the challenges of printing above 250 °C [55]. Previous attempts to reduce the costs of high-temperature 3-D printer have used retrofits of existing systems. NASA has augmented a commercial open source Lulzbot Taz (itself a RepRap) [56] and Zawaski and Williams have shown promising designs for an inverted delta-style high temperature 3-D printer [57]. To build on that previous work, in this study a Cartesian-style high-temperature 3-D printer to print PPE and other components for the COVID-19 pandemic is designed, prototyped, and validated.

## 2 Hardware description

3-D printing high temperature materials required for high-temperature heat sterilizable components and products for COVID-19 and other applications demands a 3-D printer with hot ends and heated beds that can go up to higher temperatures than the normal PTFE or even all metal hot ends on typical desktop FFF-based 3-D printers. In addition to the more thermally capable parts on the machine, the chamber in which the parts print also needs to be heated in some way to keep the parts from warping, delamination and removing themselves from the print surface. To allow for all these requirements to be met while staying under $1000, the Cerberus – a three-head RepRap was designed and released under open source licenses with the following capabilities:

• E3D high temperature heated bed (up to 200 °C) and V6 hot end (up to 500 °C) were used to allow for management of high temperature materials.
• All devices that require a relatively low operating temperature (below 70 °C) such as motors and electronics are removed from the heated print chamber to keep them from overheating.
• 1000 W space heater core is used to help the heated bed heat the chamber.
• Mains voltage is used for the chamber heater and heated bed to keep current draw low while also allowing for rapid heating times.

## 3 Design files

The design file summary is shown in Table 1.

**Table 1:** Design Files Summary.

Design file name	Image	File type	Open source license	Location of the file
E3D_Thermocouple_Board		STEP/STL	GNU General Public License (GPL) v3.0 and CERN Open Hardware License (OHL) v1.2	https://osf.io/gbjvf/
SiliconeLockRing		STEP/STL	GNU General Public License (GPL) v3.0 and CERN Open Hardware License (OHL) v1.2	https://osf.io/gbjvf/
PCB_Case		STEP/STL	GNU General Public License (GPL) v3.0 and CERN Open Hardware License (OHL) v1.2	https://osf.io/gbjvf/
LargeFunnel_R		STEP/STL	GNU General Public License (GPL) v3.0 and CERN Open Hardware License (OHL) v1.2	https://osf.io/gbjvf/
LargeFunnel_L		STEP/STL	GNU General Public License (GPL) v3.0 and CERN Open Hardware License (OHL) v1.2	https://osf.io/gbjvf/
DoorLatch_Pin		STEP/STL	GNU General Public License (GPL) v3.0 and CERN Open Hardware License (OHL) v1.2	https://osf.io/gbjvf/
DoorLatch		STEP/STL	GNU General Public License (GPL) v3.0 and CERN Open Hardware License (OHL) v1.2	https://osf.io/gbjvf/
SwitchCover2		STEP/STL	GNU General Public License (GPL) v3.0 and CERN Open Hardware License (OHL) v1.2	https://osf.io/gbjvf/
Switch_Cover		STEP/STL	GNU General Public License (GPL) v3.0 and CERN Open Hardware License (OHL) v1.2	https://osf.io/gbjvf/
CableClamp		STEP/STL	GNU General Public License (GPL) v3.0 and CERN Open Hardware License (OHL) v1.2	https://osf.io/gbjvf/
CableClamp2		STEP/STL	GNU General Public License (GPL) v3.0 and CERN Open Hardware License (OHL) v1.2	https://osf.io/gbjvf/
Extruder_Base		STEP/STL	GNU General Public License (GPL) v3.0 and CERN Open Hardware License (OHL) v1.2	https://osf.io/gbjvf/
Extruder_Arm		STEP/STL	GNU General Public License (GPL) v3.0 and CERN Open Hardware License (OHL) v1.2	https://osf.io/gbjvf/
TopBearingArm		STEP/STL	GNU General Public License (GPL) v3.0 and CERN Open Hardware License (OHL) v1.2	https://osf.io/gbjvf/
PelletFeeder_Mount		STEP/STL	GNU General Public License (GPL) v3.0 and CERN Open Hardware License (OHL) v1.2	https://osf.io/gbjvf/
Z_Motor_Mount_Plate		STEP/STL	GNU General Public License (GPL) v3.0 and CERN Open Hardware License (OHL) v1.2	https://osf.io/gbjvf/
LCD_Cover		STEP/STL	GNU General Public License (GPL) v3.0 and CERN Open Hardware License (OHL) v1.2	https://osf.io/gbjvf/
DoorSwitchSensorTrigger		STEP/STL	GNU General Public License (GPL) v3.0 and CERN Open Hardware License (OHL) v1.2	https://osf.io/gbjvf/
DoorSwitchSensorMount		STEP/STL	GNU General Public License (GPL) v3.0 and CERN Open Hardware License (OHL) v1.2	https://osf.io/gbjvf/
DoorHinge2		STEP/STL	GNU General Public License (GPL) v3.0 and CERN Open Hardware License (OHL) v1.2	https://osf.io/gbjvf/
DoorHinge1		STEP/STL	GNU General Public License (GPL) v3.0 and CERN Open Hardware License (OHL) v1.2	https://osf.io/gbjvf/
FanMount_MIR		STEP/STL	GNU General Public License (GPL) v3.0 and CERN Open Hardware License (OHL) v1.2	https://osf.io/gbjvf/
FanMount		STEP/STL	GNU General Public License (GPL) v3.0 and CERN Open Hardware License (OHL) v1.2	https://osf.io/gbjvf/
Fan_Inlet_Shroud		STEP/STL	GNU General Public License (GPL) v3.0 and CERN Open Hardware License (OHL) v1.2	https://osf.io/gbjvf/
FanShroudTube		STEP/STL	GNU General Public License (GPL) v3.0 and CERN Open Hardware License (OHL) v1.2	https://osf.io/gbjvf/
FanShroud		STEP/STL	GNU General Public License (GPL) v3.0 and CERN Open Hardware License (OHL) v1.2	https://osf.io/gbjvf/
PulleyCleat2		STEP/STL	GNU General Public License (GPL) v3.0 and CERN Open Hardware License (OHL) v1.2	https://osf.io/gbjvf/
BearingSupport		STEP/STL	GNU General Public License (GPL) v3.0 and CERN Open Hardware License (OHL) v1.2	https://osf.io/gbjvf/
TurntableMountV2		STEP/STL	GNU General Public License (GPL) v3.0 and CERN Open Hardware License (OHL) v1.2	https://osf.io/gbjvf/
TurntableLockRing		STEP/STL	GNU General Public License (GPL) v3.0 and CERN Open Hardware License (OHL) v1.2	https://osf.io/gbjvf/
GT2_TurntablePulley		STEP/STL	GNU General Public License (GPL) v3.0 and CERN Open Hardware License (OHL) v1.2	https://osf.io/gbjvf/
ToolBlank		STEP/STL	GNU General Public License (GPL) v3.0 and CERN Open Hardware License (OHL) v1.2	https://osf.io/gbjvf/
E3D_Clamp		STEP/STL	GNU General Public License (GPL) v3.0 and CERN Open Hardware License (OHL) v1.2	https://osf.io/gbjvf/
FanPelletMount-Copy		STEP/STL	GNU General Public License (GPL) v3.0 and CERN Open Hardware License (OHL) v1.2	https://osf.io/gbjvf/
Pellet_LockRing		STEP/STL	GNU General Public License (GPL) v3.0 and CERN Open Hardware License (OHL) v1.2	https://osf.io/gbjvf/
FanPelletMount		STEP/STL	GNU General Public License (GPL) v3.0 and CERN Open Hardware License (OHL) v1.2	https://osf.io/gbjvf/
Tool_Probe		STEP/STL	GNU General Public License (GPL) v3.0 and CERN Open Hardware License (OHL) v1.2	https://osf.io/gbjvf/
FanShroud_Pellet		STEP/STL	GNU General Public License (GPL) v3.0 and CERN Open Hardware License (OHL) v1.2	https://osf.io/gbjvf/
Tool_Middle		STEP/STL	GNU General Public License (GPL) v3.0 and CERN Open Hardware License (OHL) v1.2	https://osf.io/gbjvf/
HeatsinkFanShroud		STEP/STL	GNU General Public License (GPL) v3.0 and CERN Open Hardware License (OHL) v1.2	https://osf.io/gbjvf/
PelletFunnel		STEP/STL	GNU General Public License (GPL) v3.0 and CERN Open Hardware License (OHL) v1.2	https://osf.io/gbjvf/
HeaterFanShroud		STEP/STL	GNU General Public License (GPL) v3.0 and CERN Open Hardware License (OHL) v1.2	https://osf.io/gbjvf/
SpoolHolder		STEP/STL	GNU General Public License (GPL) v3.0 and CERN Open Hardware License (OHL) v1.2	https://osf.io/gbjvf/
Rear_Panel		STEP/STL	GNU General Public License (GPL) v3.0 and CERN Open Hardware License (OHL) v1.2	https://osf.io/gbjvf/
ElectronicsPanel		STEP/STL	GNU General Public License (GPL) v3.0 and CERN Open Hardware License (OHL) v1.2	https://osf.io/gbjvf/
BottomPanel		STEP/STL	GNU General Public License (GPL) v3.0 and CERN Open Hardware License (OHL) v1.2	https://osf.io/gbjvf/
Left_Side_Panel		STEP/STL	GNU General Public License (GPL) v3.0 and CERN Open Hardware License (OHL) v1.2	https://osf.io/gbjvf/
FrontPanel		STEP/STL	GNU General Public License (GPL) v3.0 and CERN Open Hardware License (OHL) v1.2	https://osf.io/gbjvf/
DoorTrim		STEP/STL	GNU General Public License (GPL) v3.0 and CERN Open Hardware License (OHL) v1.2	https://osf.io/gbjvf/
SideDoorTrim		STEP/STL	GNU General Public License (GPL) v3.0 and CERN Open Hardware License (OHL) v1.2	https://osf.io/gbjvf/
Firewall_Panel		STEP/STL	GNU General Public License (GPL) v3.0 and CERN Open Hardware License (OHL) v1.2	https://osf.io/gbjvf/
HotendInsulationPlate		STEP/STL	GNU General Public License (GPL) v3.0 and CERN Open Hardware License (OHL) v1.2	https://osf.io/gbjvf/
Rotary_Encoder_wheel		STEP/STL	GNU General Public License (GPL) v3.0 and CERN Open Hardware License (OHL) v1.2	https://osf.io/gbjvf/
Sensor_Base		STEP/STL	GNU General Public License (GPL) v3.0 and CERN Open Hardware License (OHL) v1.2	https://osf.io/gbjvf/
SensorMount		STEP/STL	GNU General Public License (GPL) v3.0 and CERN Open Hardware License (OHL) v1.2	https://osf.io/gbjvf/
MotorMount		STEP/STL	GNU General Public License (GPL) v3.0 and CERN Open Hardware License (OHL) v1.2	https://osf.io/gbjvf/
BedBeltMount		STEP/STL	GNU General Public License (GPL) v3.0 and CERN Open Hardware License (OHL) v1.2	https://osf.io/gbjvf/
Z_SidePlates		STEP/STL	GNU General Public License (GPL) v3.0 and CERN Open Hardware License (OHL) v1.2	https://osf.io/gbjvf/
Z_Axis_Mid_Plate		STEP/STL	GNU General Public License (GPL) v3.0 and CERN Open Hardware License (OHL) v1.2	https://osf.io/gbjvf/
x_Angle_Iron		STEP/STL	GNU General Public License (GPL) v3.0 and CERN Open Hardware License (OHL) v1.2	https://osf.io/gbjvf/
Middle_Panel_Pulley		STEP/STL	GNU General Public License (GPL) v3.0 and CERN Open Hardware License (OHL) v1.2	https://osf.io/gbjvf/
BeltTransferPlate		STEP/STL	GNU General Public License (GPL) v3.0 and CERN Open Hardware License (OHL) v1.2	https://osf.io/gbjvf/
20mm_Angle_Iron		STEP/STL	GNU General Public License (GPL) v3.0 and CERN Open Hardware License (OHL) v1.2	https://osf.io/gbjvf/
X_Axis_EndPlate		STEP/STL	GNU General Public License (GPL) v3.0 and CERN Open Hardware License (OHL) v1.2	https://osf.io/gbjvf/
AluminumPrintBedSupport		STEP/STL	GNU General Public License (GPL) v3.0 and CERN Open Hardware License (OHL) v1.2	https://osf.io/gbjvf/
Bed_X_EndCap		STEP/STL	GNU General Public License (GPL) v3.0 and CERN Open Hardware License (OHL) v1.2	https://osf.io/gbjvf/
BearingAngleIron		STEP/STL	GNU General Public License (GPL) v3.0 and CERN Open Hardware License (OHL) v1.2	https://osf.io/gbjvf/
Z_Motor_Mount		STEP/STL	GNU General Public License (GPL) v3.0 and CERN Open Hardware License (OHL) v1.2	https://osf.io/gbjvf/
E3d_Mount		STEP/STL	GNU General Public License (GPL) v3.0 and CERN Open Hardware License (OHL) v1.2	https://osf.io/gbjvf/
Repetier-Firmware_Chamber	Firmware for two hotends, heated bed, and heated chamber.	Repetier.ino	GNU General Public License (GPL) v3.0 and CERN Open Hardware License (OHL) v1.2	https://osf.io/gbjvf/
Slicer Settings	Full Slicer profiles for materials and print settings. In HighTemp\Documentation\Software. The best profiles are for Cura in Repetier Host.	.rcp	GNU General Public License (GPL) v3.0 and CERN Open Hardware License (OHL) v1.2	https://osf.io/gbjvf/

STEP files are included for editing and STL for printing. The Repetier.ino firmware file is for two hotends, a heated bed and a heated chamber. In addition, the Cura engine slicer configuration files for Repetier Host for 3-D printing the final high temperature materials is included.

### 3.1 Bill of materials

The BOMs are shown in Table 2, Table 3, Table 4.

**Table 2:** Bill of Materials.

Order Source	Part Name	Unit Quantity	Rounded to Package Quantity	Unit Price	Price
	**HARDWARE & FRAMING**				
ZYLTech	90°Corner	86	7	$4.95	$34.65
ZYLTech	8 mm M4 Button Head	292	3	$4.95	$14.85
ZYLTech	M4 Extrusion Nuts	292	3	$9.95	$29.85
ZYLTech	T-Slot L Inside 90°	10	1	$4.95	$4.95
ZYLTech	2020–20 Extrusion	41	2	$53.99	$107.98
Amazon	WJB 6810-2RS	1	2	$15.49	$30.98
McMaster Carr	3/4in Aluminum L angle		1	$7.07	$7.07
Amazon	Aluminum 215x215 plate	1	1	$19.99	$19.99
McMaster Carr	Aluminum 1/8in thick plate	1	1	$9.42	$9.42
Amazon	Tooth Idler Pulleys 3 mm	5	1	$5.99	$5.99
Amazon	Smooth Idler Pulleys 3 mm bore	4	1	$5.99	$5.99
Local Hardware Store	Panel Material-1/4in birch plywood	N/A	1	$28.00	$28.00
Amazon	Brass Insert Set	N/A	1	$19.00	$19.00
Amazon	Standoffs	6	1	$9.79	$9.79
Amazon	Flanged Bearings	2	1	$10.68	$10.68
E3D	Gates GT2 Belt	1	1	$8.00	$8.00
Amazon	M3 Thumb Nuts	4	1	$5.20	$5.20
Amazon	Mid load springs	4	1	$7.59	$7.59
Amazon	5 mm rod	1	1	$6.44	$6.44

	**LINEAR MOTION**				
ZYLTech	MGN12 Rail Single Block	2	2	$24.95	$49.90
ZYLTech	MGN12 Rail Double Block	2	2	$34.95	$69.90
ZYLTech	SBR16 Linear Rail Set	1	1	$34.95	$34.95

	**ELECTRONICS**				
E3D	Nema 17 Stepper Motor Unipolar L = 48 mm w/ Gear Ratio 14:1 Planetary Gearbox	1	1	$29.99	$29.99
E3D	Nema17 High Torque Motor (E3D)	3	3	$12.60	$37.80
E3D	Nema 17 External 48 mm Stack 0.4A Lead 2 mm/0.07874″ Length 330 mm	1	1	$33.99	$33.99
E3D	High Temperature Heated Bed (E3D)	1	1	$107.25	$107.25
Amazon	Solid State Relays	1	1	$9.56	$9.56
Amazon	Borosilicate Glass (E3D)	1	1	$11.44	$11.44
E3D	Swiss Clips (E3D)	4	4	$0.47	$1.88
Amazon	RAMPS 1.4 w/ full graphics disp	1	1	$35.99	$35.99
Amazon	Power Supply 12 V	1	1	$25	$25
Amazon	Brushless Radial Fan	1	1	$17.58	$17.58
Amazon	PTC Ceramic Air Heater 110 V/220 V 1000 W	1	1	$28.79	$28.79
Amazon	Microswitch	4	1	$7	$7
	Blue Sea Systems A-Series Toggle Single Pole Circuit Breakers	1	1	$15	$15

	**E3D V6 High Temp Hotend**				
E3D	E3D V6	1	1	$55.62	$55.62
E3D	Thermocouple Kit	1	1	$28	$28
E3D	High Temperature Heater Cartridge	1	1	$62.09	$62.09
E3D	Stepper Motor	1	1	$12	$12
Amazon	Extruder Gear	1	1	$3	$3
Amazon	Bowden tube and fittings	1	1	$8	$8

**Table 3:** 3-D printed components.

*3D Printed Parts*	*Quantity*	*Material*
E3D_Thermocouple_Board	1	PETG
SiliconeLockRing	1	PETG
PCB_Case	1	PETG
LargeFunnel_R	1	PETG
LargeFunnel_L	1	PETG
DoorLatch_Pin	1	PETG
SwitchCover2	1	PETG
Switch_Cover	1	PETG
CableClamp	1	PETG
CableClamp2	1	PETG
Extruder_Base	2	PETG
Extruder_Arm	2	PETG
TopBearingArm	1	PETG
PelletFeeder_Mount	1	PETG
Z_Motor_Mount_Plate	1	PETG
LCD_Cover	1	PETG
DoorSwitchSensorTrigger	1	PETG
DoorSwitchSensorMount	1	PETG
DoorHinge1	2	PETG
DoorHinge2	2	PETG
FanMount_MIR	1	PETG
FanMount	1	PETG
Fan_Inlet_Shroud	1	PETG
FanShroudTube	1	PETG
FanShroud	1	PETG
PulleyCleat2	1	PETG
BearingSupport	1	PETG
TurntableMountV2	1	PETG
TurntableLockRing	1	PETG
GT2_TurntablePulley	1	PETG
ToolBlank	1	PETG
E3D_Clamp	1	PETG
FanPelletMount	1	PETG
Pellet_LockRing	1	PETG
FanPelletMount	1	PETG
Tool_Probe	1	PETG
FanShroud_Pellet	1	PETG
Tool_Middle	1	PETG
HeatsinkFanShroud	1	PETG
PelletFunnel	1	PETG
HeaterFanShroud	1	PEKK
SpoolHolder	1	PETG
Rotary_Encoder_wheel	1	PETG
Sensor_Base	1	PETG
SensorMount	1	PETG
Z_Motor_Mount_Plate	1	PETG
E3d_Mount	1	PETG

The 3-D printed parts are printed in PETG on any RepRap-class FFF 3-D printer [41], [42], [43].

**Table 4:** Extrusions.

EXTRUSIONS			
Size	Source	Length	Count
20–2020	ZYLTech	600 mm	6
20–2020	ZYLTech	470 mm	14
20–2040	ZYLTech	400 mm	2
20–2020	ZYLTech	340 mm	4
20–2020	ZYLTech	350 mm	2
20–2020	ZYLTech	150 mm	2
20–2020	ZYLTech	140 mm	2
20–2020	ZYLTech	100 mm	4
20–2020	ZYLTech	60 mm	2

## 4 Build instructions

### 4.1 Printer Frame

**Fig. 1:**
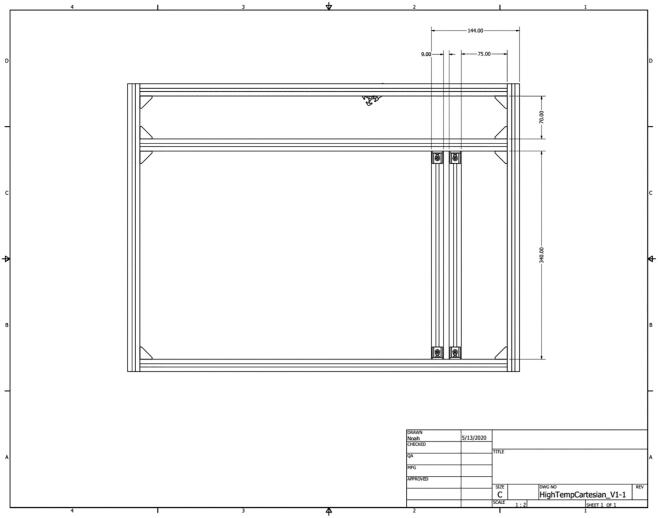
Front view of extrusions (all measurements in mm).

The listed 2020 aluminum extrusions in the bill of materials (BOM) with lengths of 470 mm and 600 mm are used to construct the front and rear of the printer as shown in Fig. 1. They are assembled using 90-degree angle brackets, M5 by 8 mm long screws, and M5 T-slot nuts. The distances between the various extrusions is also shown in Fig. 1. Three of the front sections shown should be constructed. One for the front door panel and two more for the two sides of the frame.

**Fig. 2:**
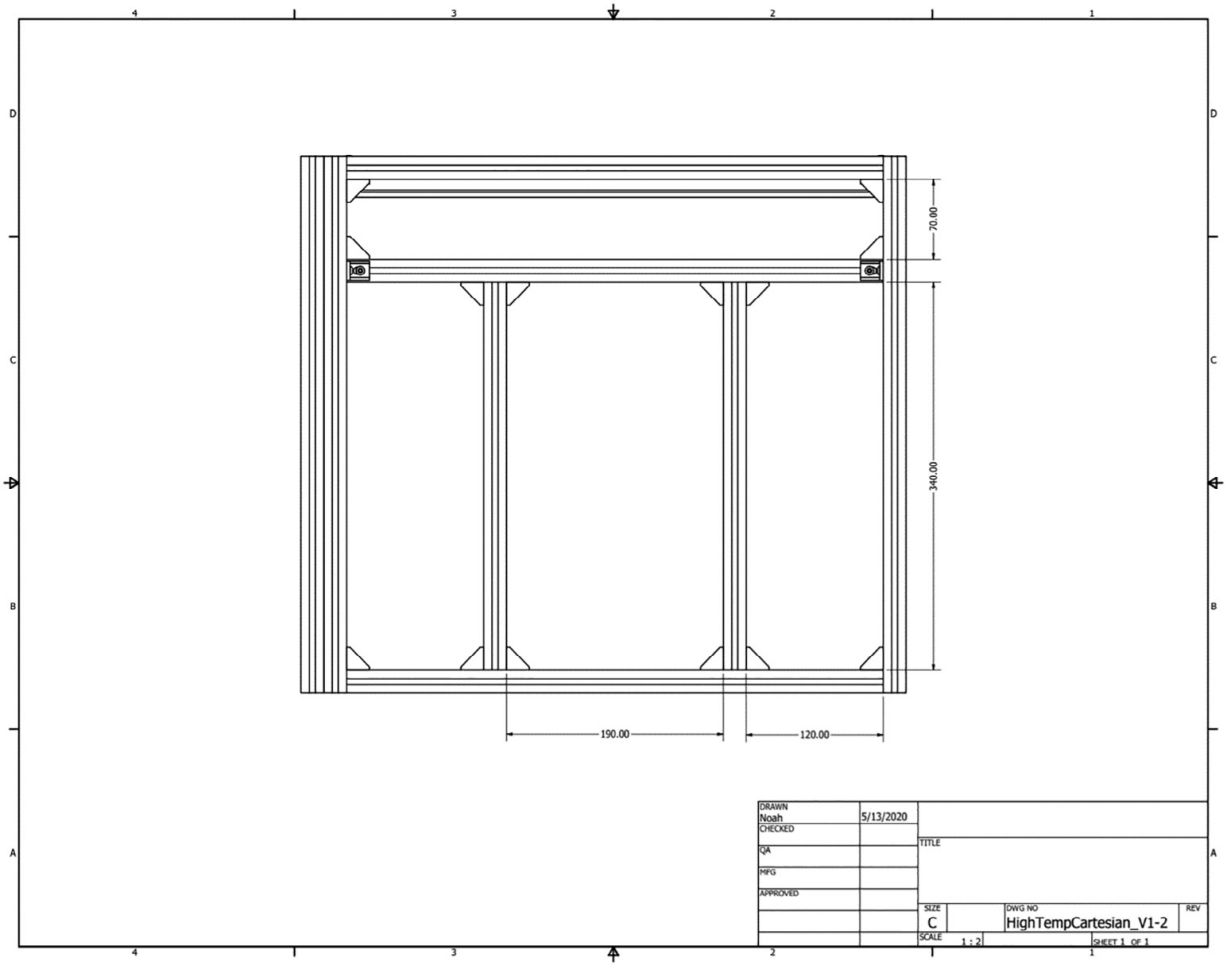
Right side view of extrusions (all measurements in mm).

**Fig. 3:**
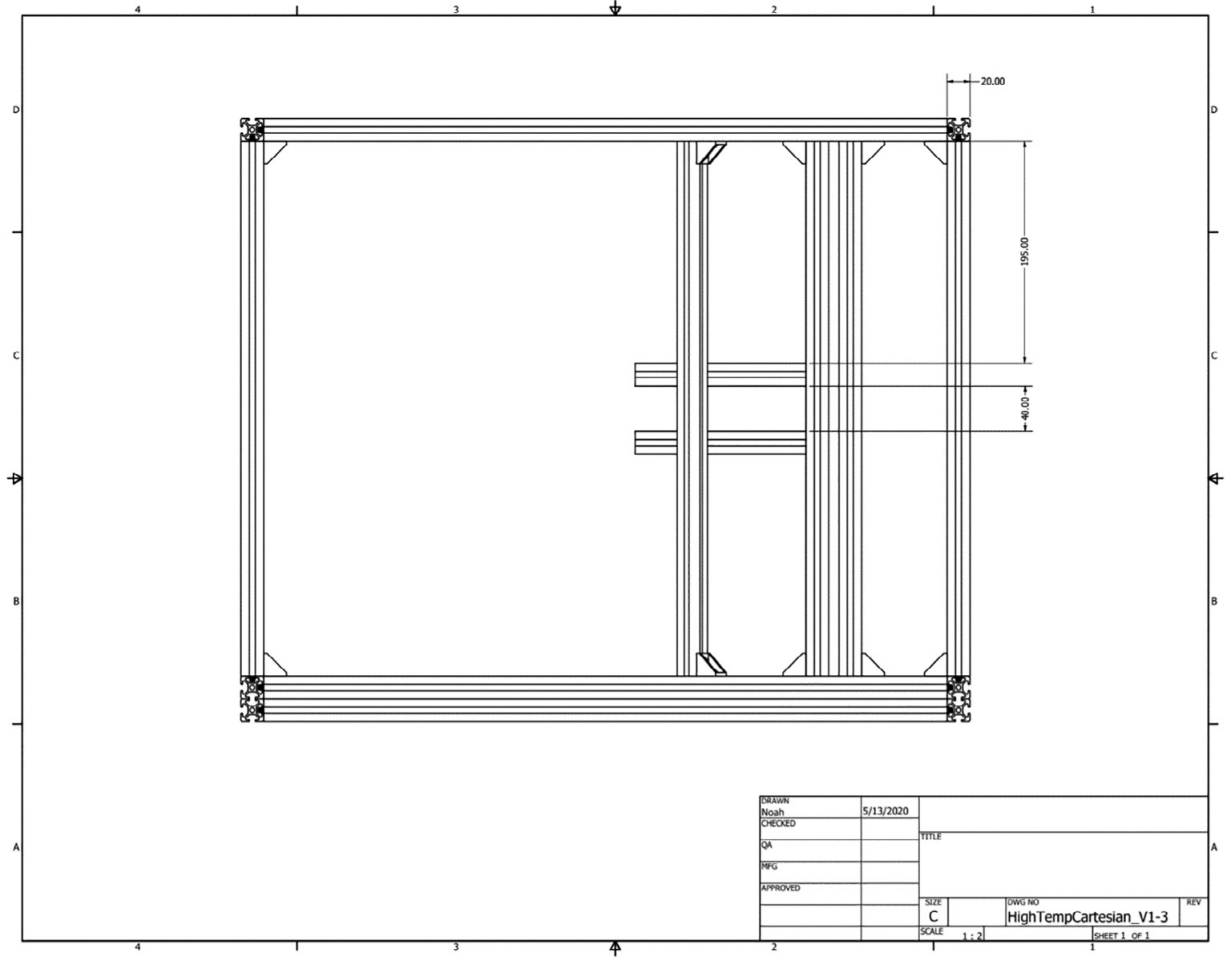
Top view of extrusions (all measurements in mm).

**Fig. 4:**
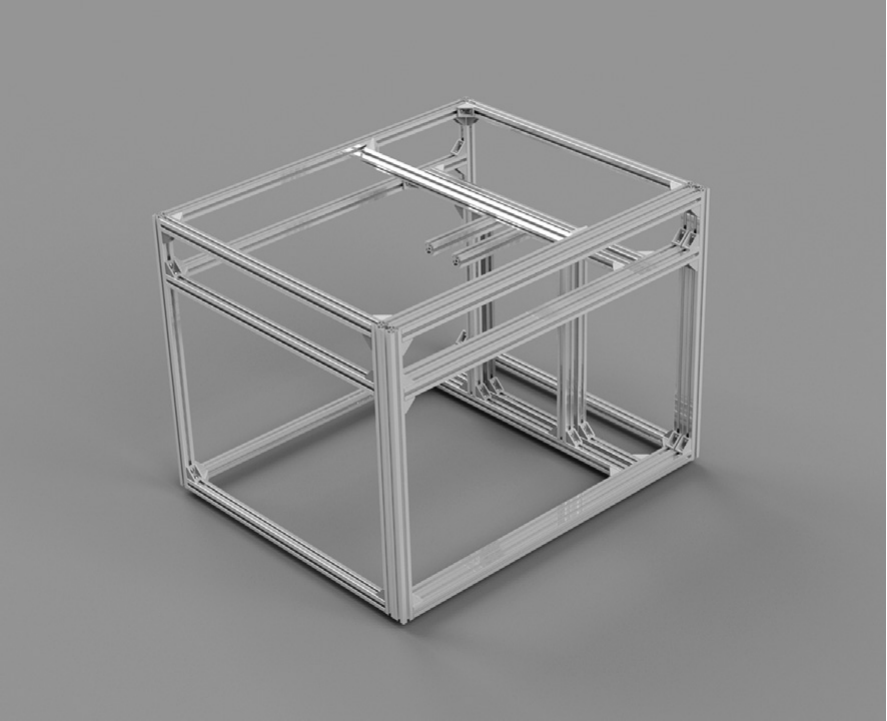
Printer Frame.

For the left and right sides of the machine, extrusions with a length of 470 mm are used to attach the front and pack sections together. The spacing between the different extrusions is shown in Fig. 1, Fig. 2. The vertical extrusions that are shown in Fig. 2, and are what hold up the Z-axis and are attached as shown in the Fig. 3. The completed frame should resemble that shown in Fig. 4.

The complete frame in Fig. 4 consists of the main frame and a hinged front door. The frame is the first overall step in the assembly process. The design of the frame is intended to be easy to modify and allow for an enclosed chamber that can be heated without overheating the electrical or 3-D printed components inside the printer. All extrusions can be cut using a hacksaw and the miter box included in the stl files for the rest of the machine. This strategy for cutting was effective if careful attention was paid to getting the extrusion lengths correct and the ends cut as square as possible.

### 4.2 Motion platform

**Fig. 5:**
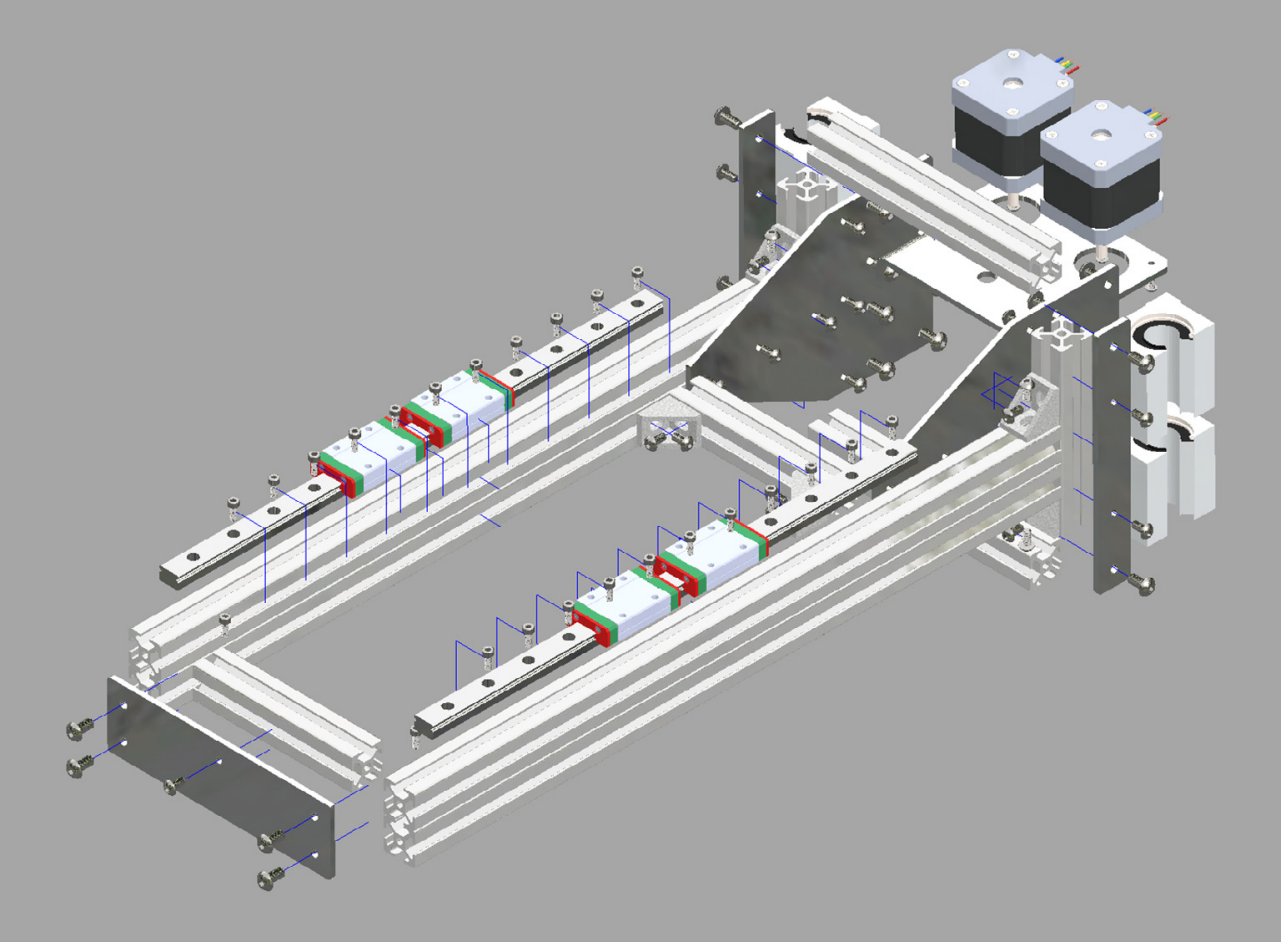
X and Z axis view.

**Fig. 6:**
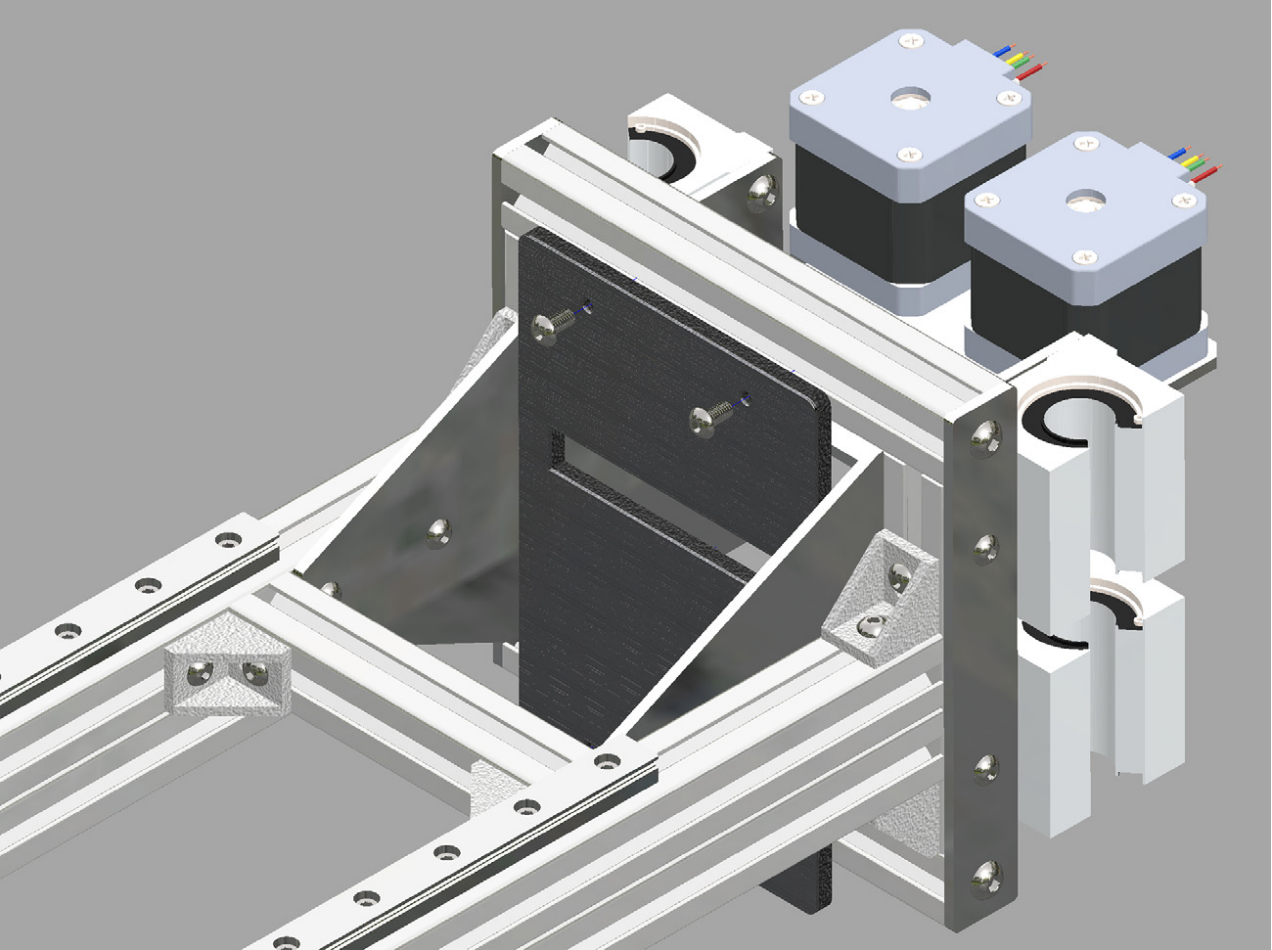
Insulation plate.

The motion platform consists of a bed that moves in both X and Y on the assembly and the entire assembly moves up and down on the Z axis rails. The print bed is the design change that allows for the high temperature chamber. The assembly process is shown in steps below. Fig. 5 shows the main section of the Z-axis. The locations for the gantry should be constrained by the two main support plates. The rails can also be attached at this point, but cannot be tightened until the other axis is attached to ensure that the rails stay square to each other. The 16 mm vertical rail bearings also have to be aligned in this way. Fabricators should insert the large rail section into the two bearings before tightening down the bolts that hold it together. Fig. 6 shows the insulation plate that helps keep as much heat inside the system as possible.

**Fig. 7:**
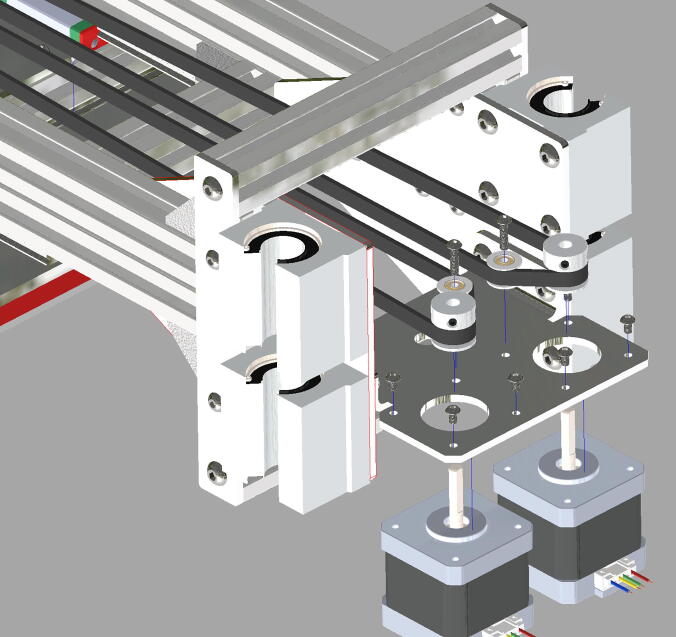
Motor and pulley mounts.

The two motors that control the x and y axes movements are each attached using three screws that hold the motor on and one for holding the belt idler pulley tight. To keep the tensioners tight, a longer bolt is used that tightens into the thread limit inside the motors (about 7 mm into the motor). It is recommended that the motor wires be turned outwards for easy access during the wiring steps. It is also possible to add motor noise isolators by using a M3 nut to act as a jam nut for the idler pulleys. Fig. 7 shows this assembly.

**Fig. 8:**
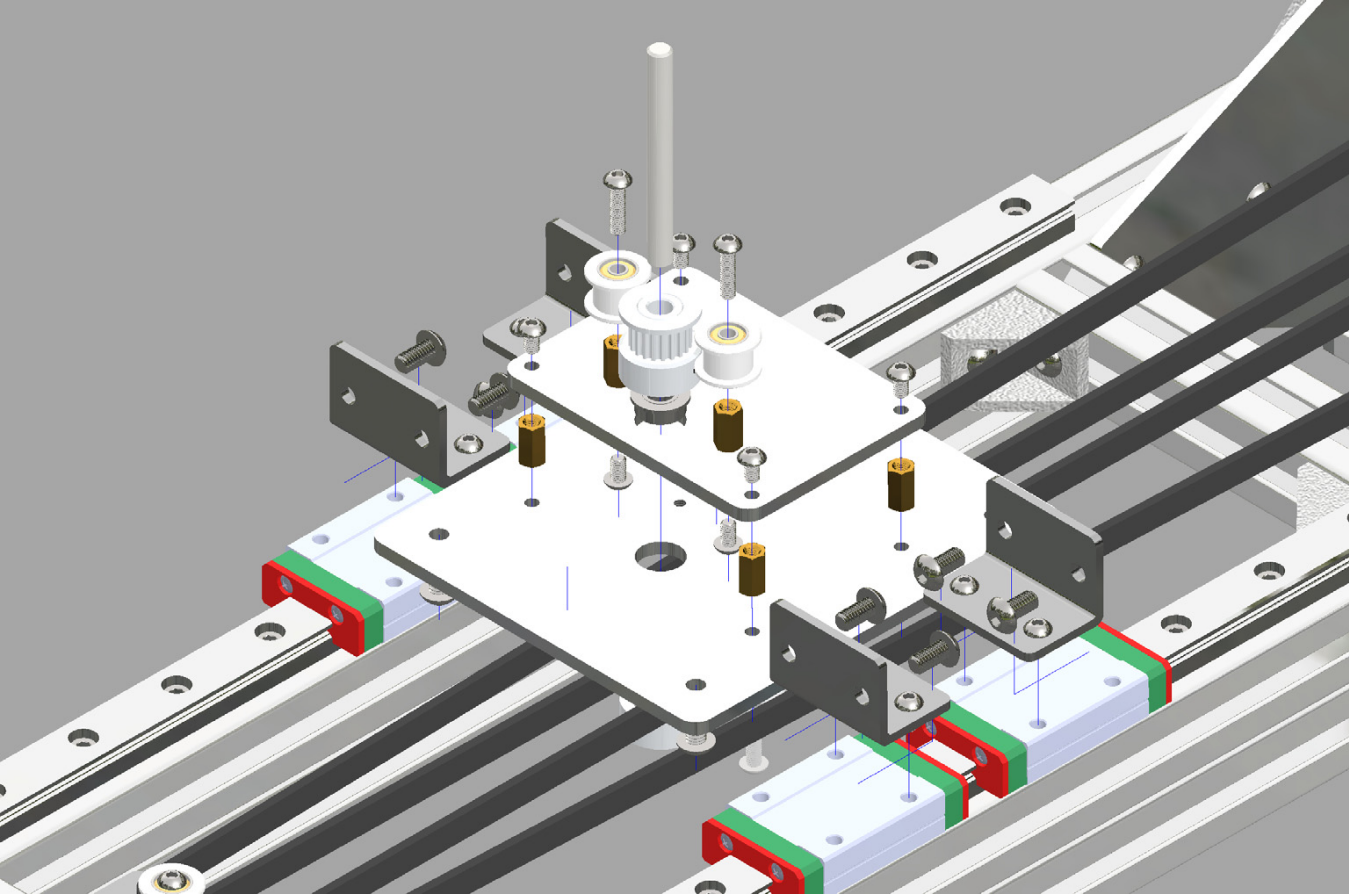
X to Y belt transmission side 1.

**Fig. 9:**
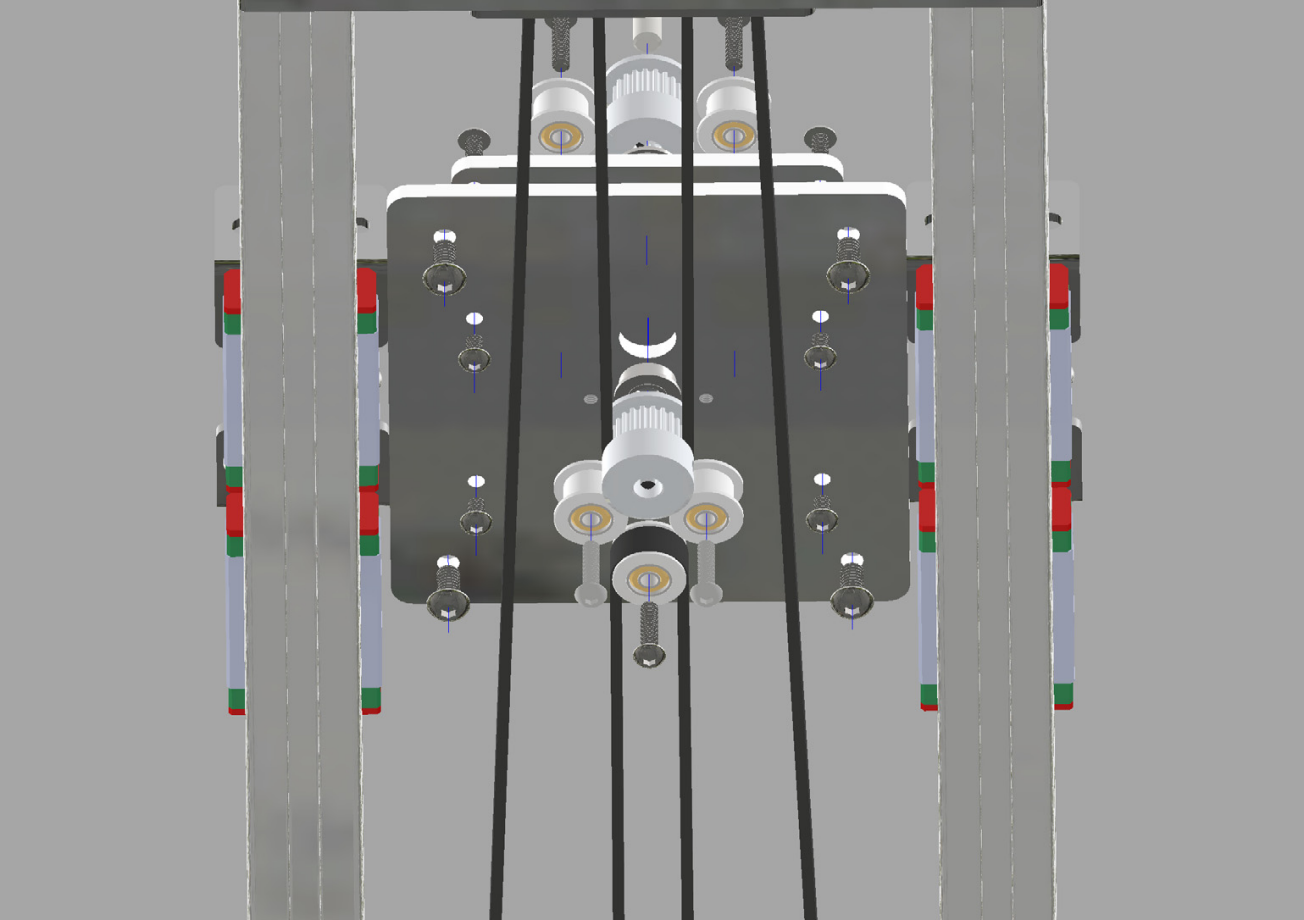
X to Y belt transmission side 2.

Another important section to make this assembly operational is the x to y transmission. Rotating the center pulley controls the y axis and pulling and pushing on the lower pulleys controls the x axis movement. Fig. 8 shows a top view of this assembly and Fig. 9 shows the lower view of the assembly.

**Fig. 10:**
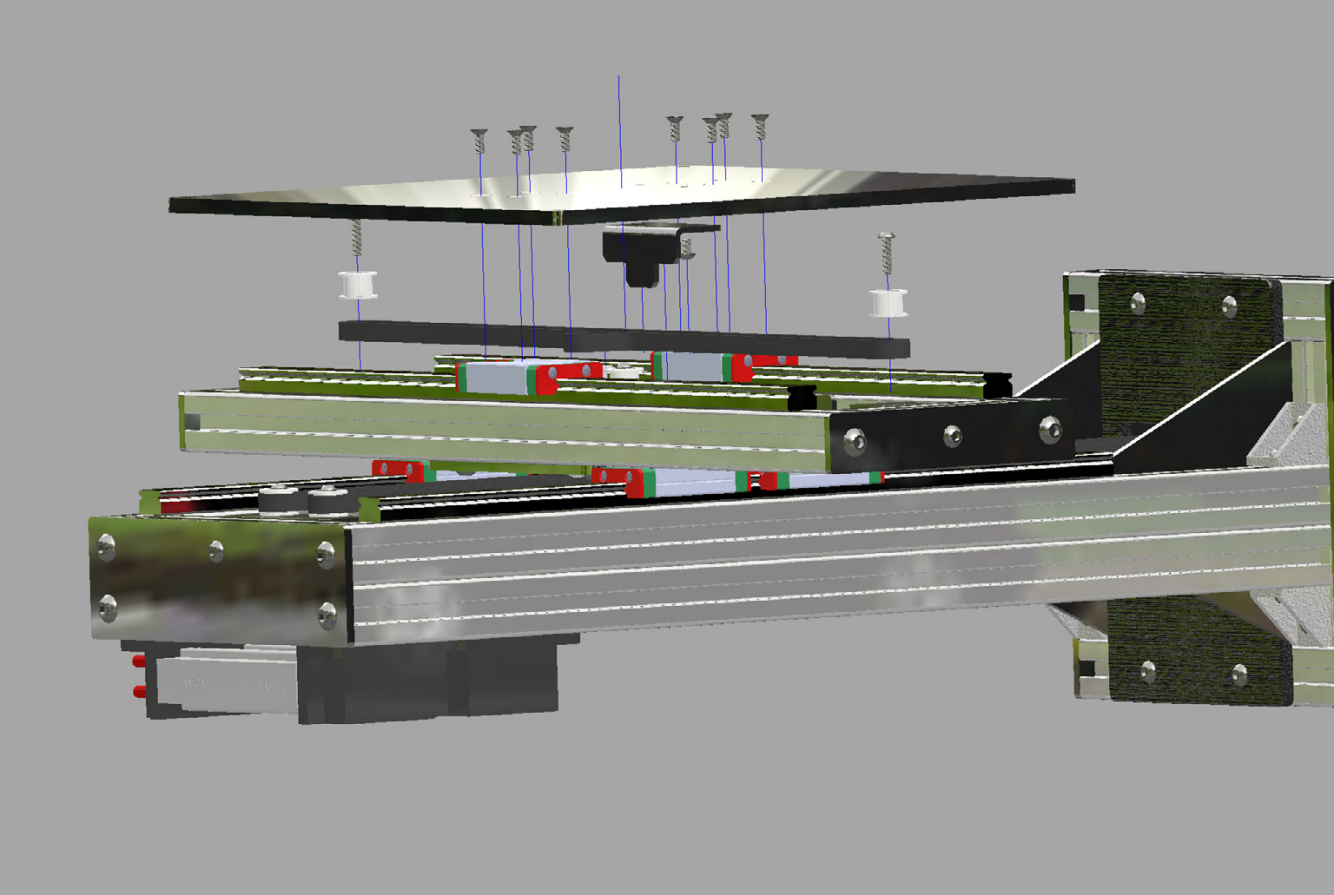
Y axis assembly.

**Fig. 11:**
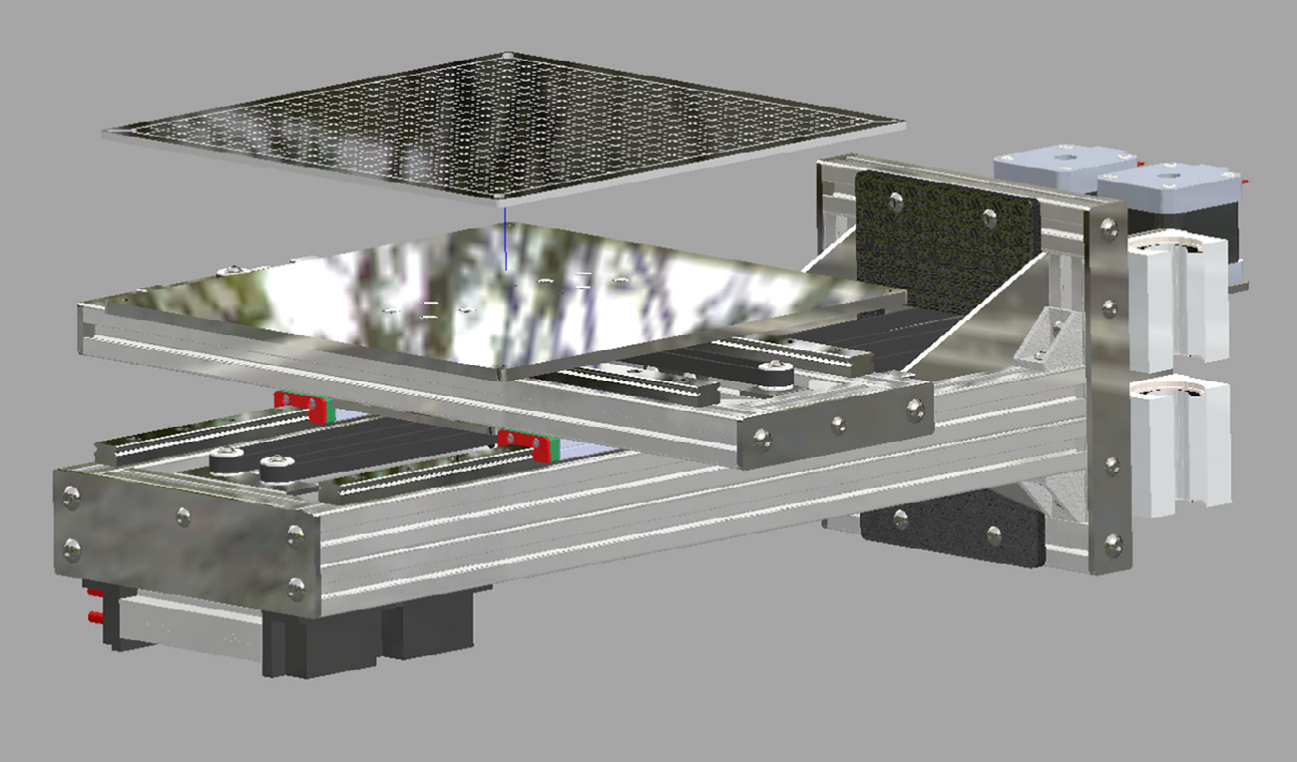
Heated bed mounting.

Next, the y axis is added to the system. As with the x axis, the y axis must be aligned by keeping the bolts on the rail loose and then slide the other sliders back and forth to ensure that it does not bind before tightening down the bolts. The belt should be mounted before the bed support plate is mounted. This is shown in Fig. 10. Once this is complete the heated bed can be mounted to the support plate. This is done using 25 mm–30 mm M3 screws and bed spring with a thumb screw below the plate to allow for manual bed leveling of the system. Fig. 11 shows the heated bed being mounted.

**Fig. 12:**
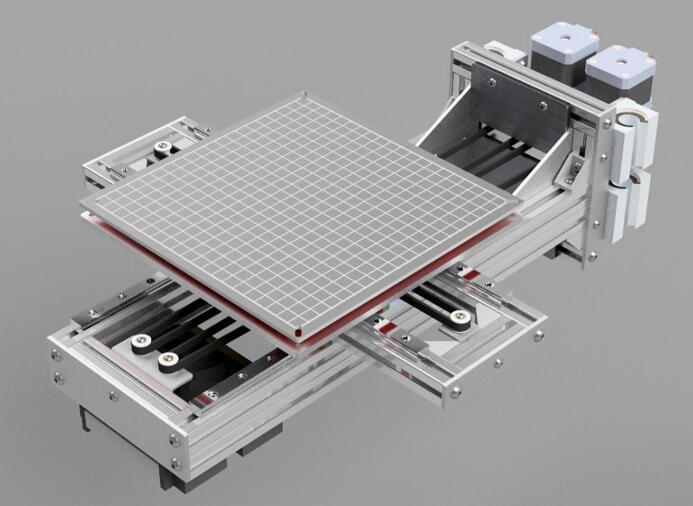
Completed motion platform assembly.

This completes the assembly of the main motion platform. The motion of the motors in relation to the bed resembles that of a CoreXY [58] where moving only one motor will move the bed diagonally one way and moving the other will move it diagonally the other way. The bed should be able to move relatively freely and without too much vibration. If it does, it is possible to realign the bearings to make it smoother. If this does not work, fabricators should check the belt line and make sure it does not rub on anything and it is tight. Lastly one can clean out the bearings and re-grease them with some thin grease. The completed assembly is shown in Fig. 12.

### 4.3 Rotary tool head

**Fig. 13:**
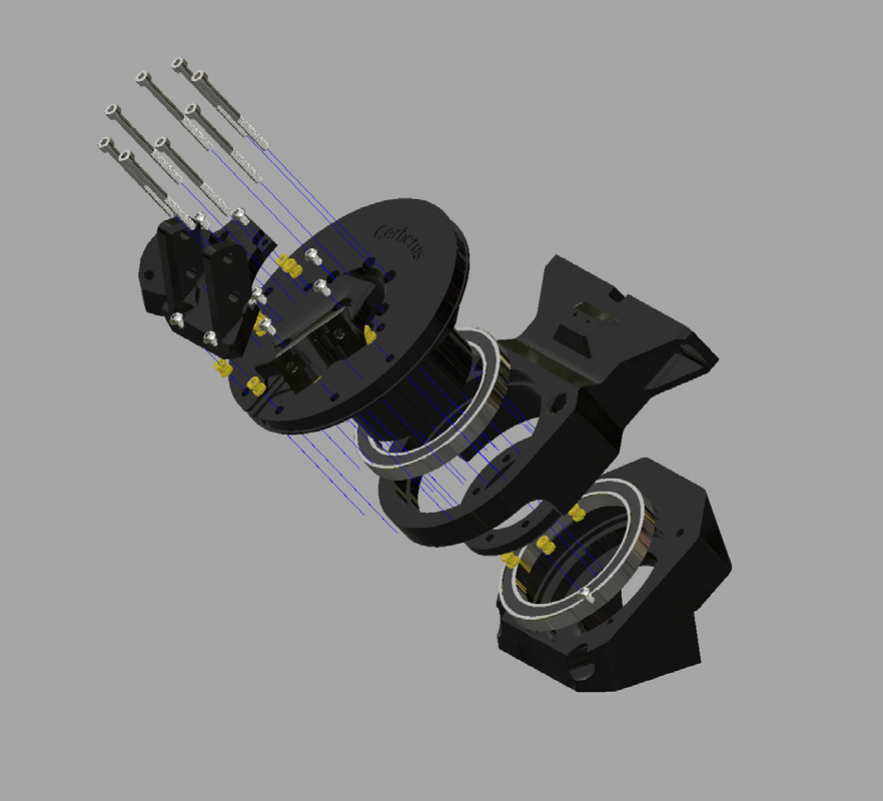
Rotary tool head assembly V1.

The design for the motion platform allows for any tool head to be added at any scale without influencing the motion platform and motion print quality. To take advantage of this, a rotary tool head was utilized to both allow for high temperature auto bed leveling and bed probing and multiple other tool heads controlled by a stepper motor. Fig. 13 shows the first step in assembling the turntable and the tools that were used in the initial tests of the machine.

**Fig. 14:**
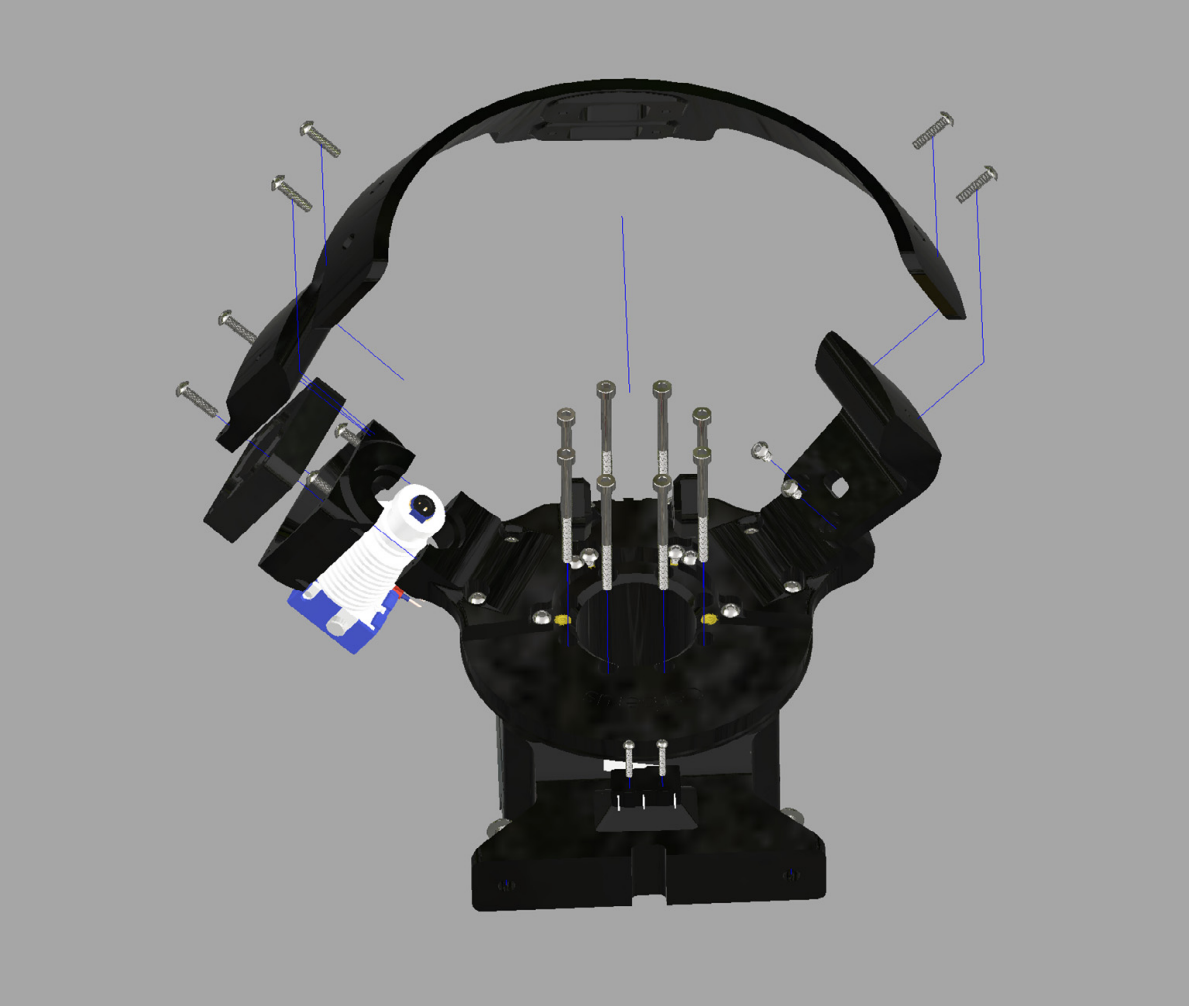
Rotary Tool with E3D V6 and probe.

Fig. 14 shows the next step in assembling the tool head. The large ring that is attached to the outside is meant to support the tool head and a slot for a potential pellet extruder. The center bolts attach to a lock ring behind the last bearing to lock it all together.

### 4.4 Printer assembly

**Fig. 15:**
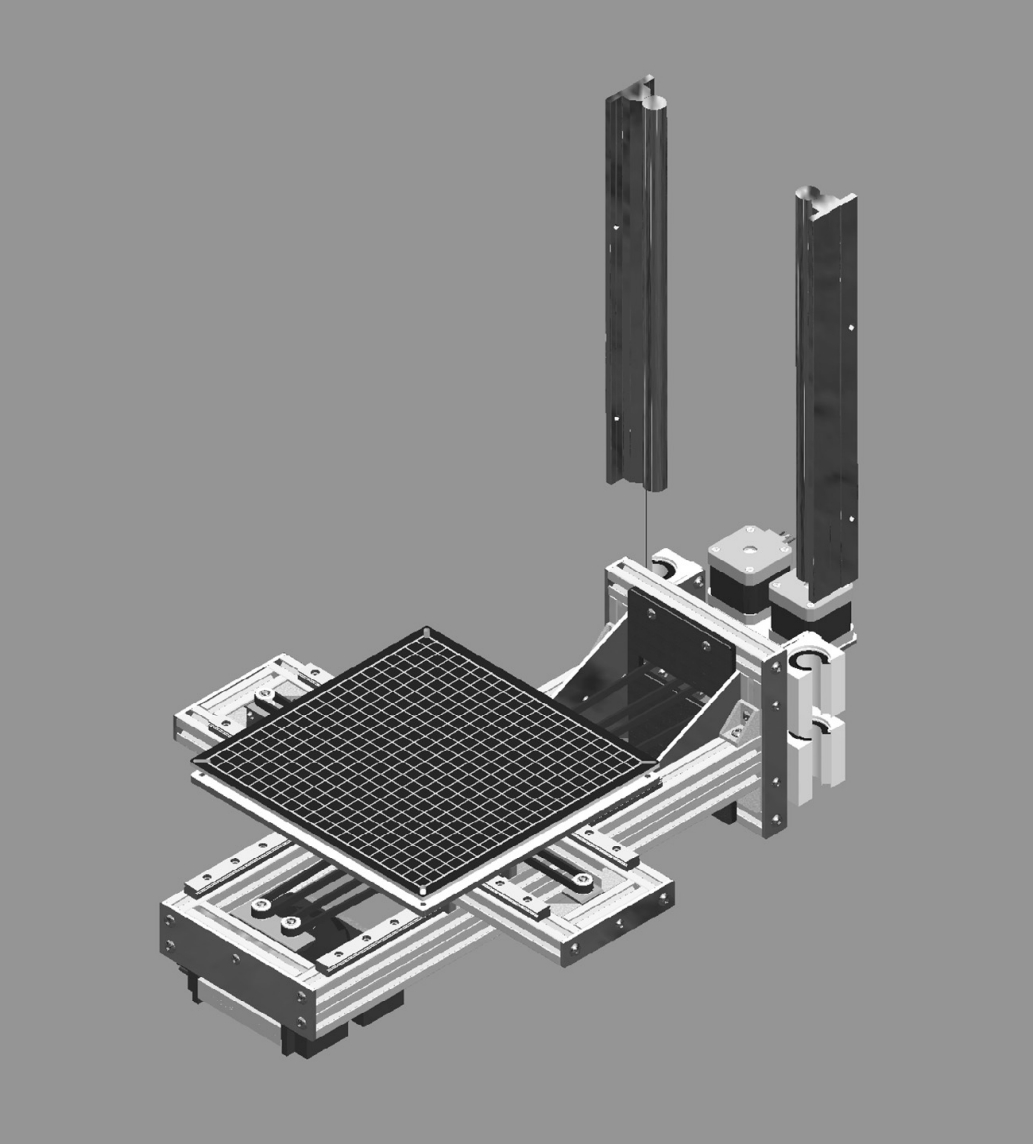
Z rail installation.

**Fig. 16:**
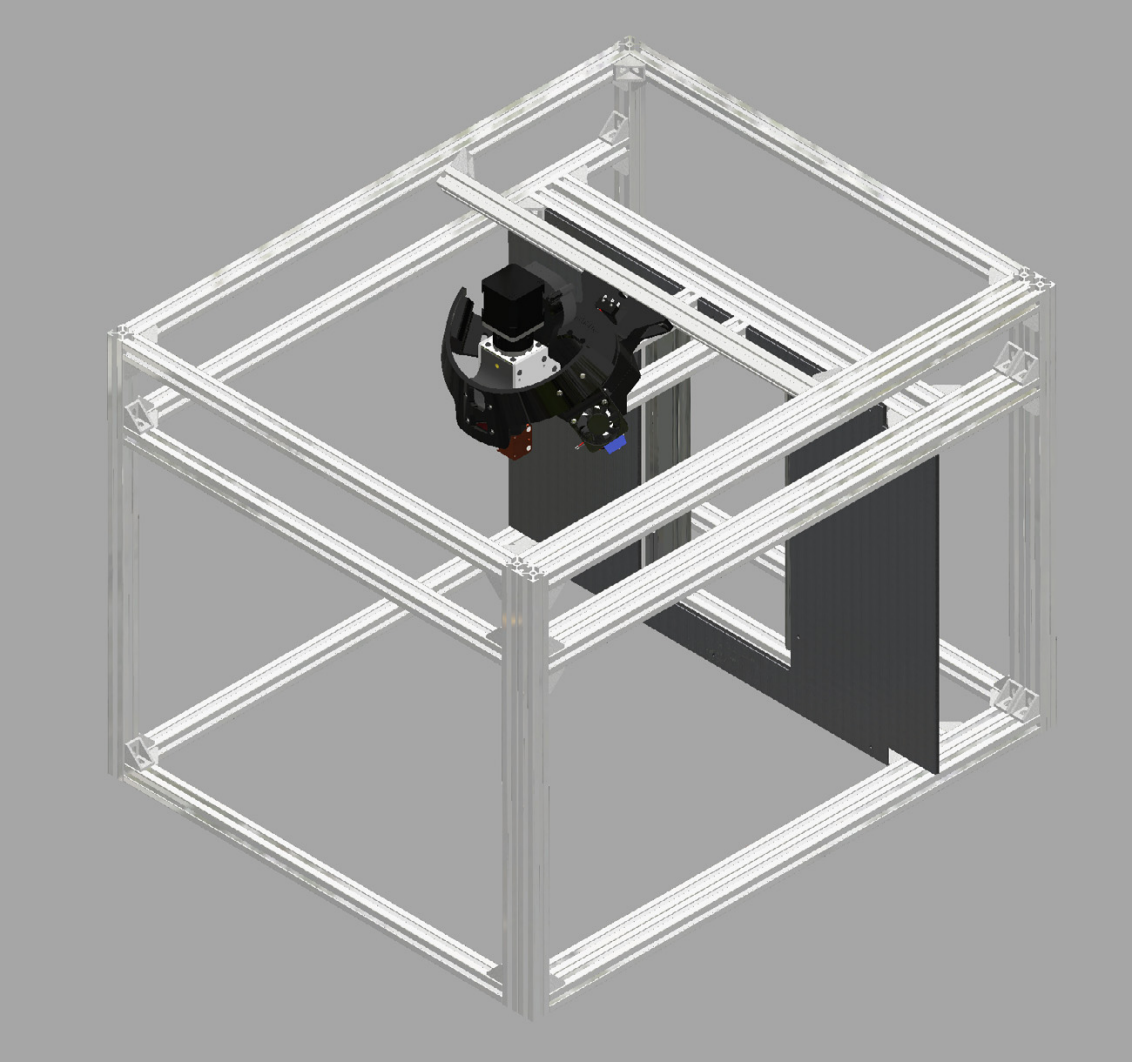
Firewall and tool head installed.

**Fig. 17:**
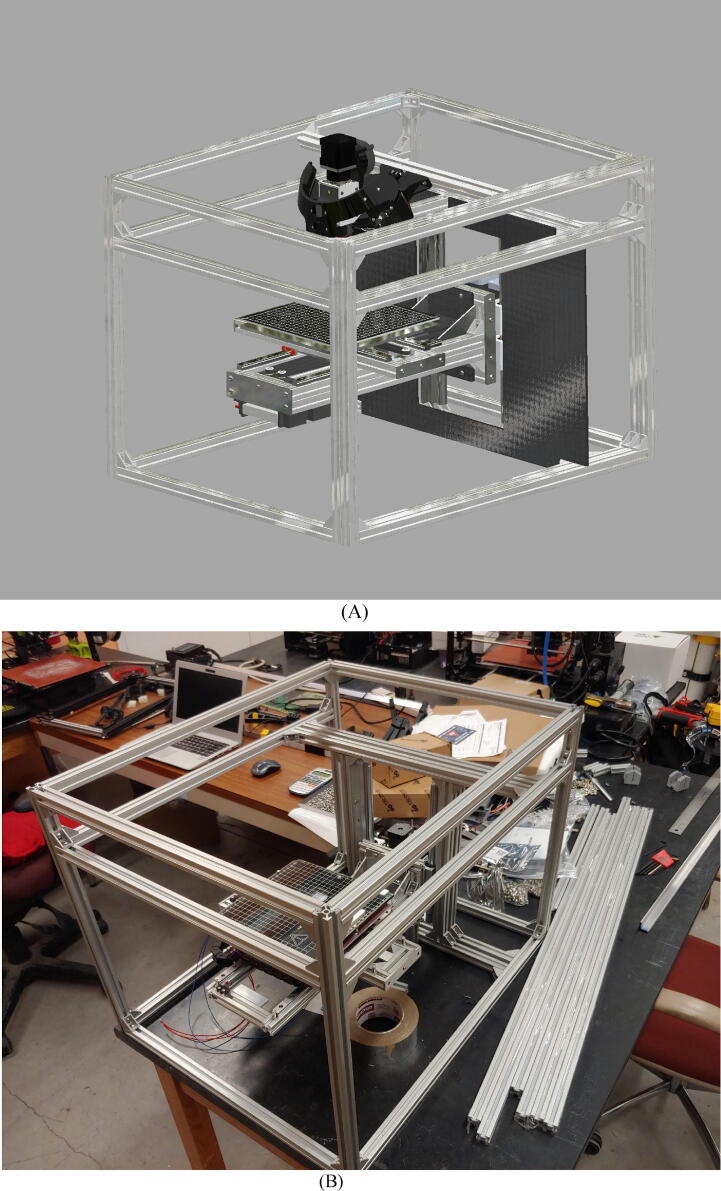
(A) Z gantry installed and installation test without firewall (B).

Now the three subassemblies can be combined together and assembled into a 3-D printer. The first step in this process is to insert the 16 mm Z-axis rails into the previously assembled Z gantry. This process is shown in Fig. 15. The tool head mounts using four bolts, two M6 bolts into the ends of the two extrusions reaching for the center of the machine and the other two are M4 that attach to a cross beam on the top of the printer. This beam is at an angle and this is intended to match the angle of the tool head. Before the installation of the z gantry, the firewall panel must be installed. 10 mm M4 bolts and T-Slot nuts are used for this. Both steps are shown in Fig. 16. The rails and the Z gantry can then be bolted into place using eight M4 or M5 bolts and T-Slot nuts into the vertical rails as shown in Fig. 17.

**Fig. 18:**
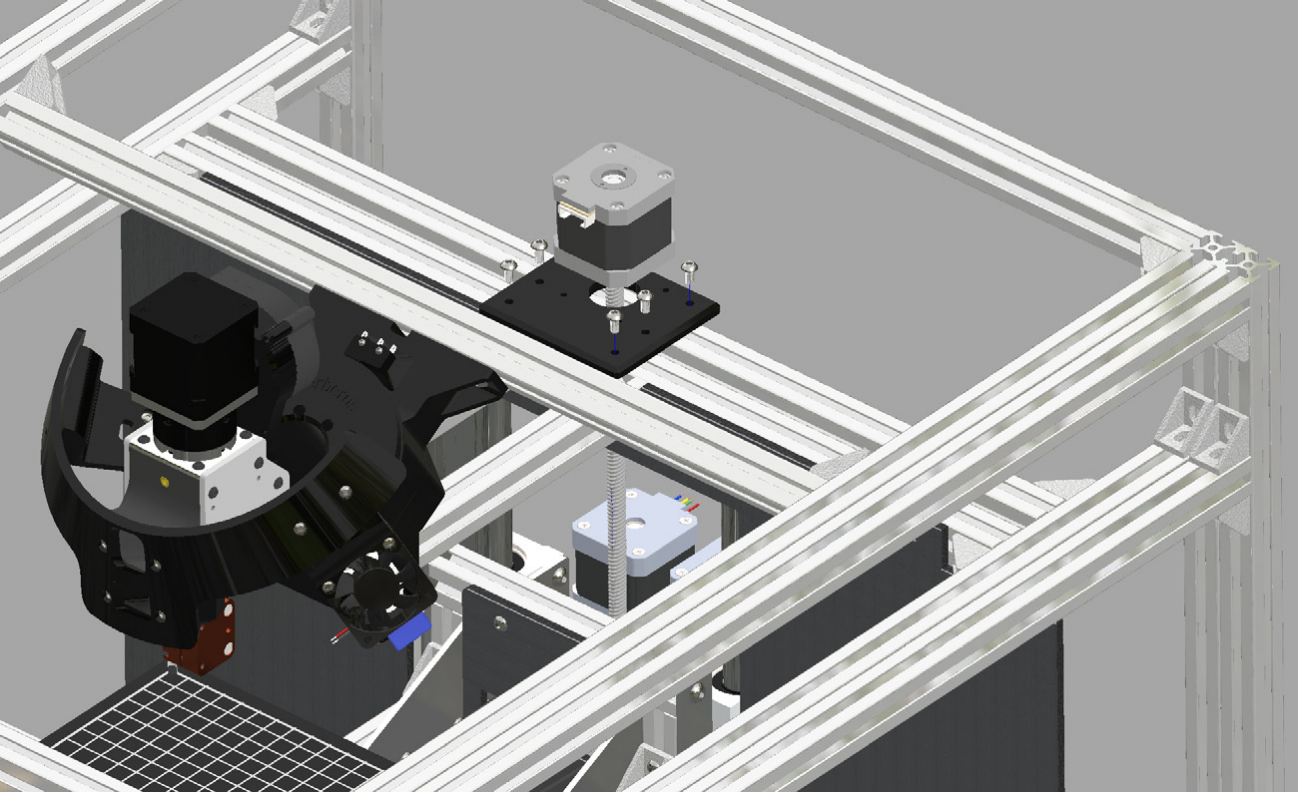
Z motor installation.

To move the z axis up and down, a NEMA 17 integrated leadscrew motor is installed on the top and the nut is bolted to the plate the x and y motors are attached to. The motor bolts to the plate first and then that plate is bolted to the frame as shown in Fig. 18.

**Fig. 19:**
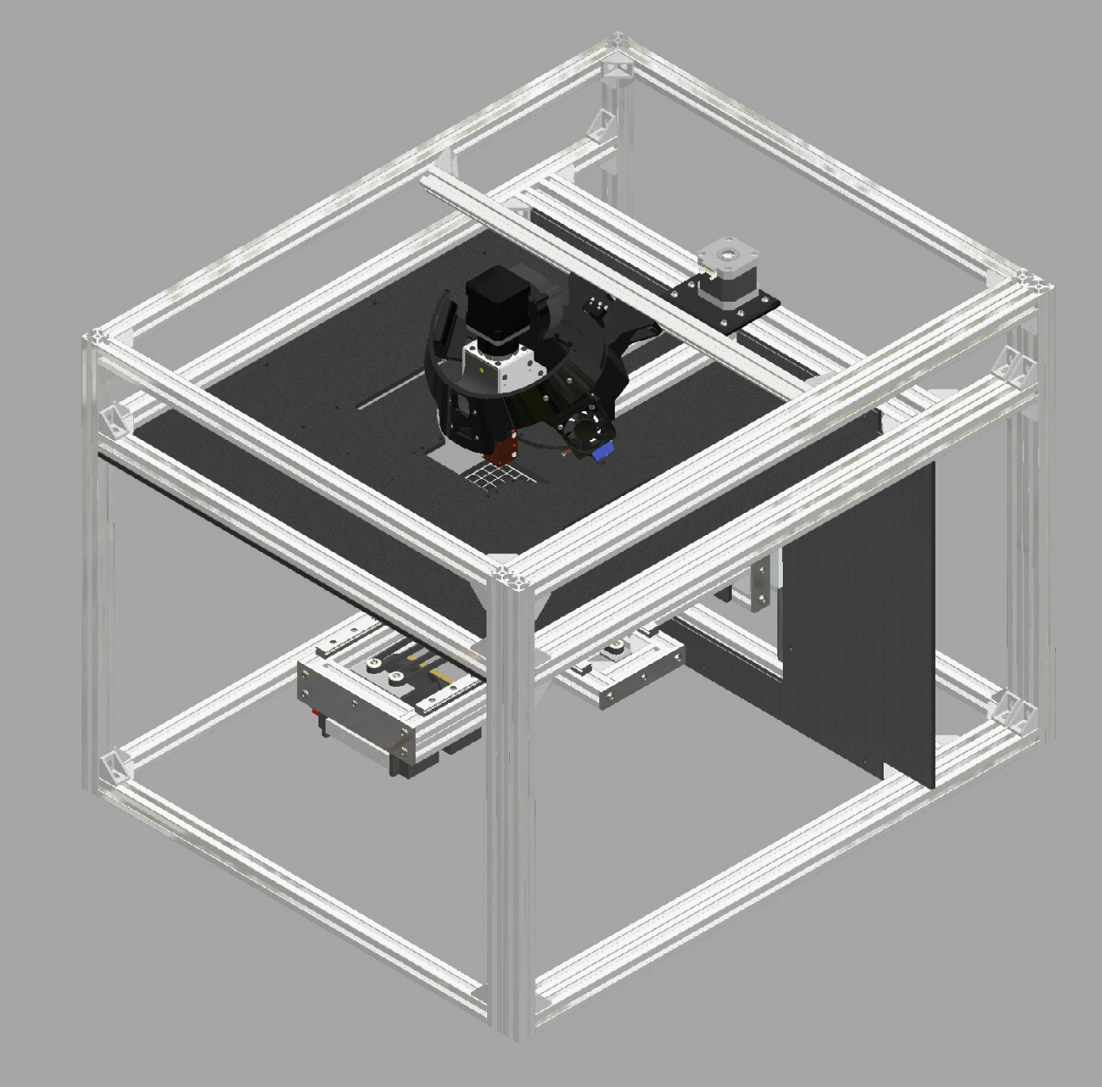
Tool head insulation installation.

**Fig. 20:**
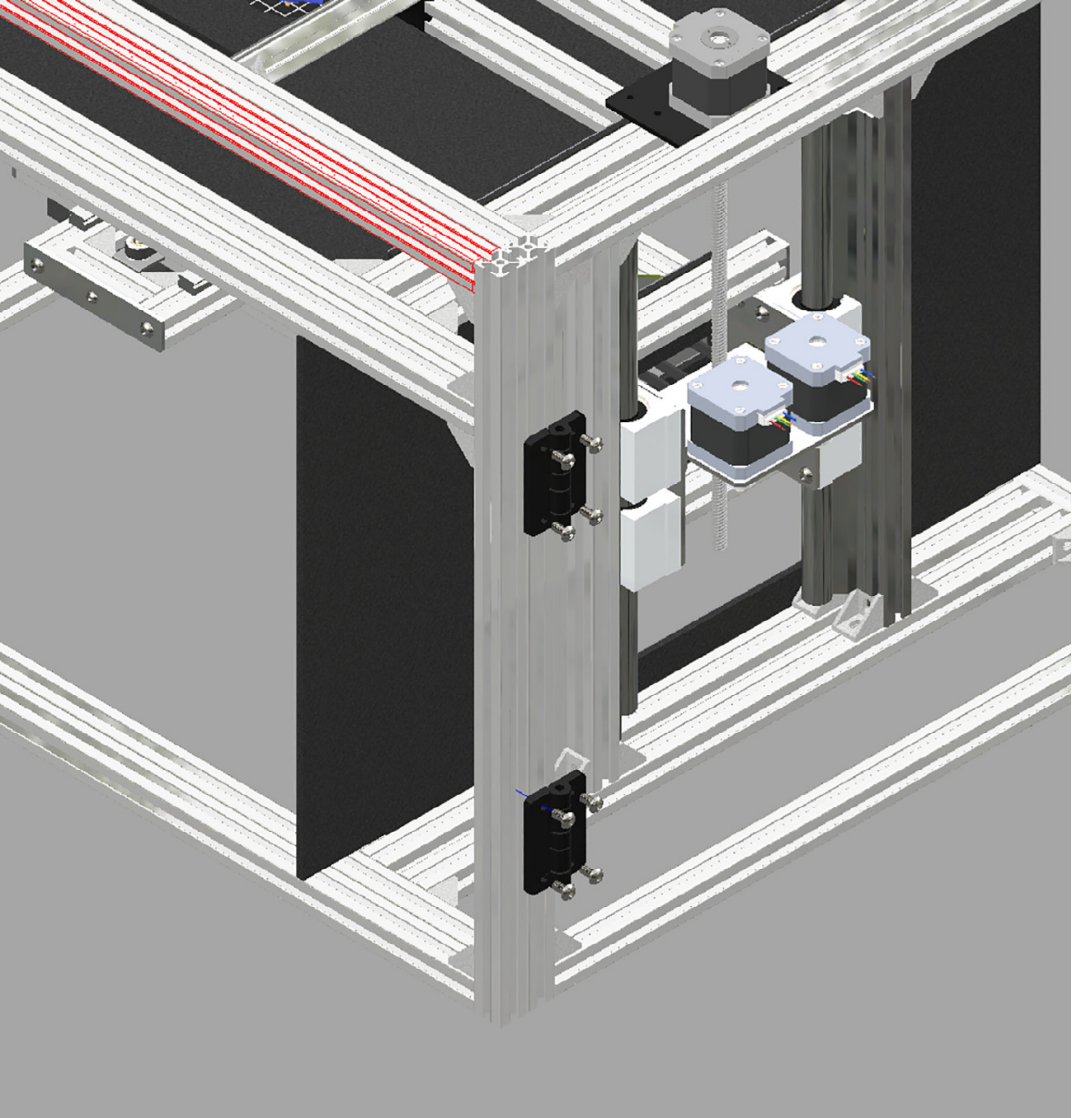
Hinge attachment.

After these steps, the paneling can be attached. In this case, the panels were laser cut out of 5 mm thick birch plywood and painted black. The inside of the paneling was covered with a thin insulation film that was glued to the panels using spray adhesive. The most complicated panel to install is the top tool head insulation plate. This plate is bolted to the frame in the same manner that the firewall was. It goes underneath the secondary frame extrusions. This is shown in Fig. 19. The front extrusions also need hinges before the next steps, so those are installed next as shown in Fig. 20 using eight 8 mm M4 bolts and T-slot nuts. The positions of the hinges are determined by the rear electronics panel cutouts.

**Fig. 21:**
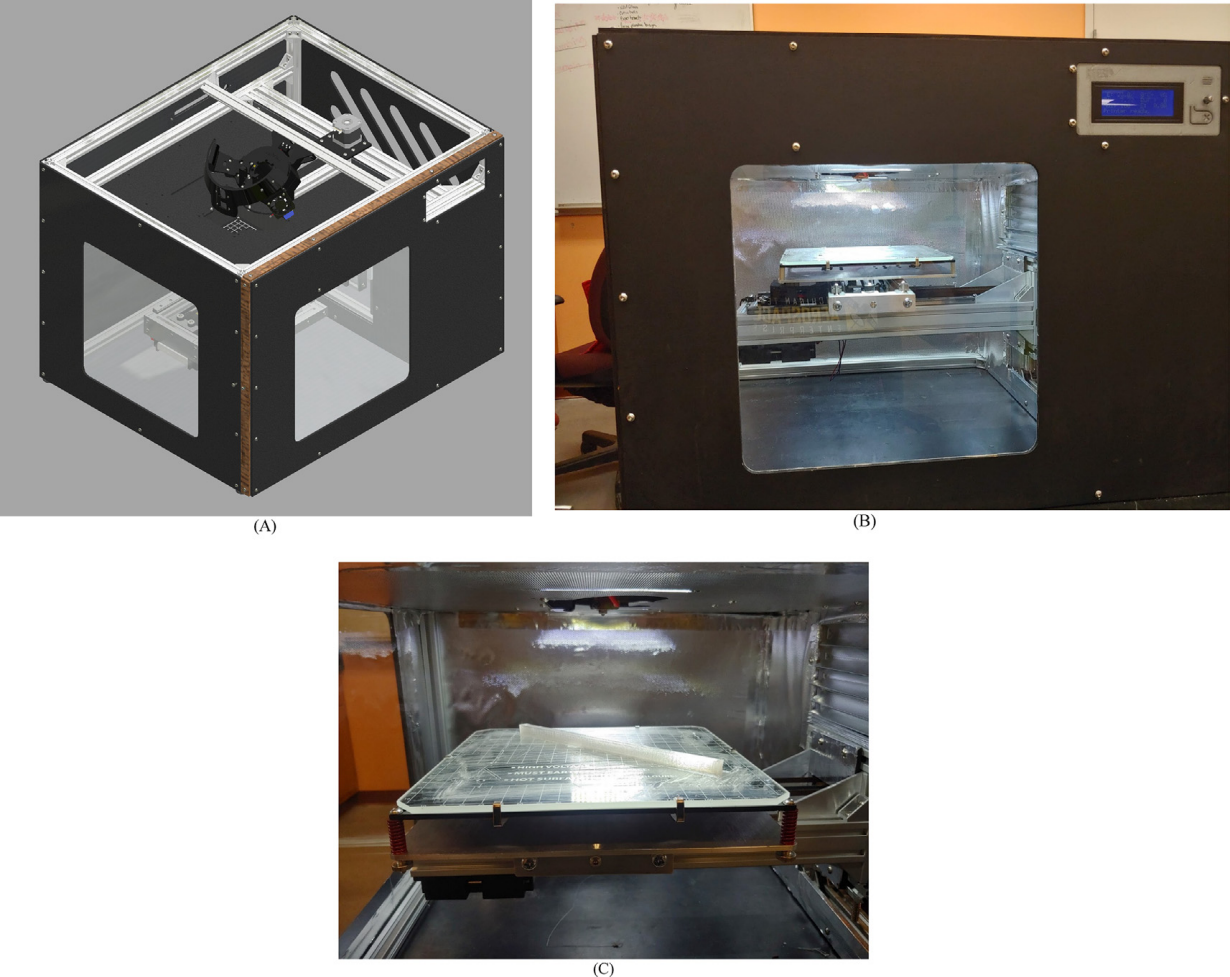
(A) Panel installation, (B) inside sealed with aluminum tape in and (C) with test print on bed.

Next, the panels are all be installed. The orientations and positions for the panels are shown in Fig. 21. Before installing the panels, the insulation must be adhered to the panels as mentioned with the top panel. This step is not necessary, but helps with the performance of the heated chamber and longevity of the panels on the outside. All panels are attached using M4 by 8 mm long bolts and T-slot nuts. The inside of the panels at the joints are sealed closed using aluminum tape along all the seams. Aluminum tape is also used to seal 12in glass sheets to the panels along with some adhesive. The top panel is left off to allow for easier access to the wiring and tool head for assembly.

**Fig. 22:**
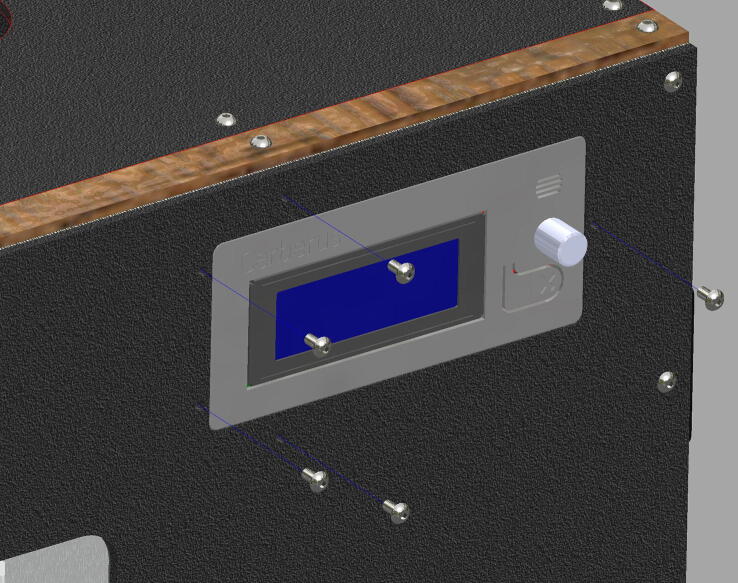
Screen assembly.

At this point the screen can be attached using the 3-D printed mount and screen. A few M4 bolts hold it together as shown in Fig. 22. This screen will eventually need longer extension cables to reach the RAMPS board. The printer does support SD card printing and the SD card can be reached by opening the door and installing the SD card.

**Fig. 23:**
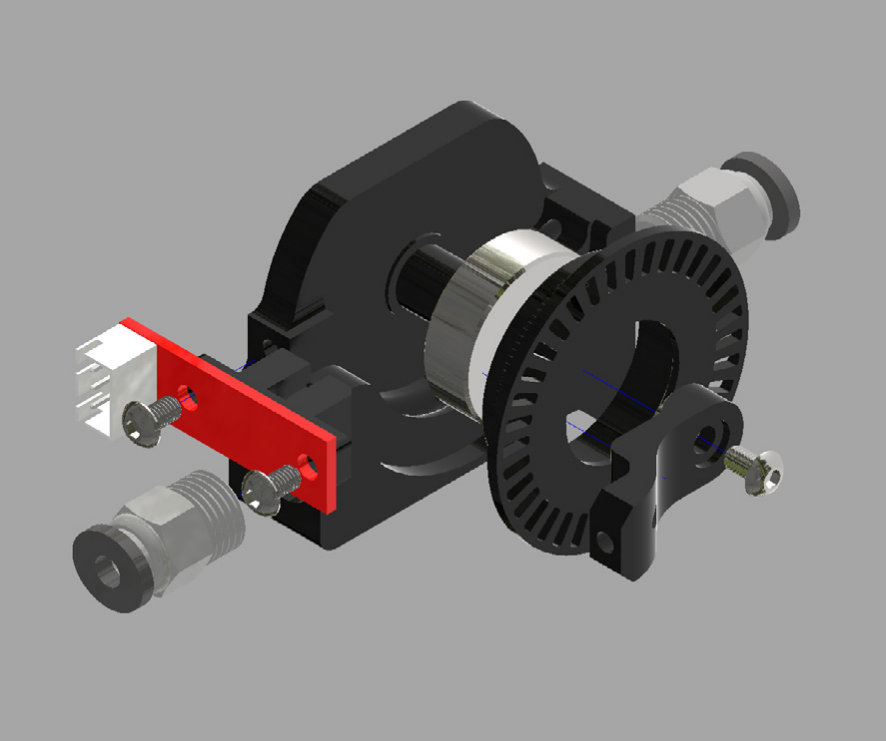
Filament sensor.

**Fig. 24:**
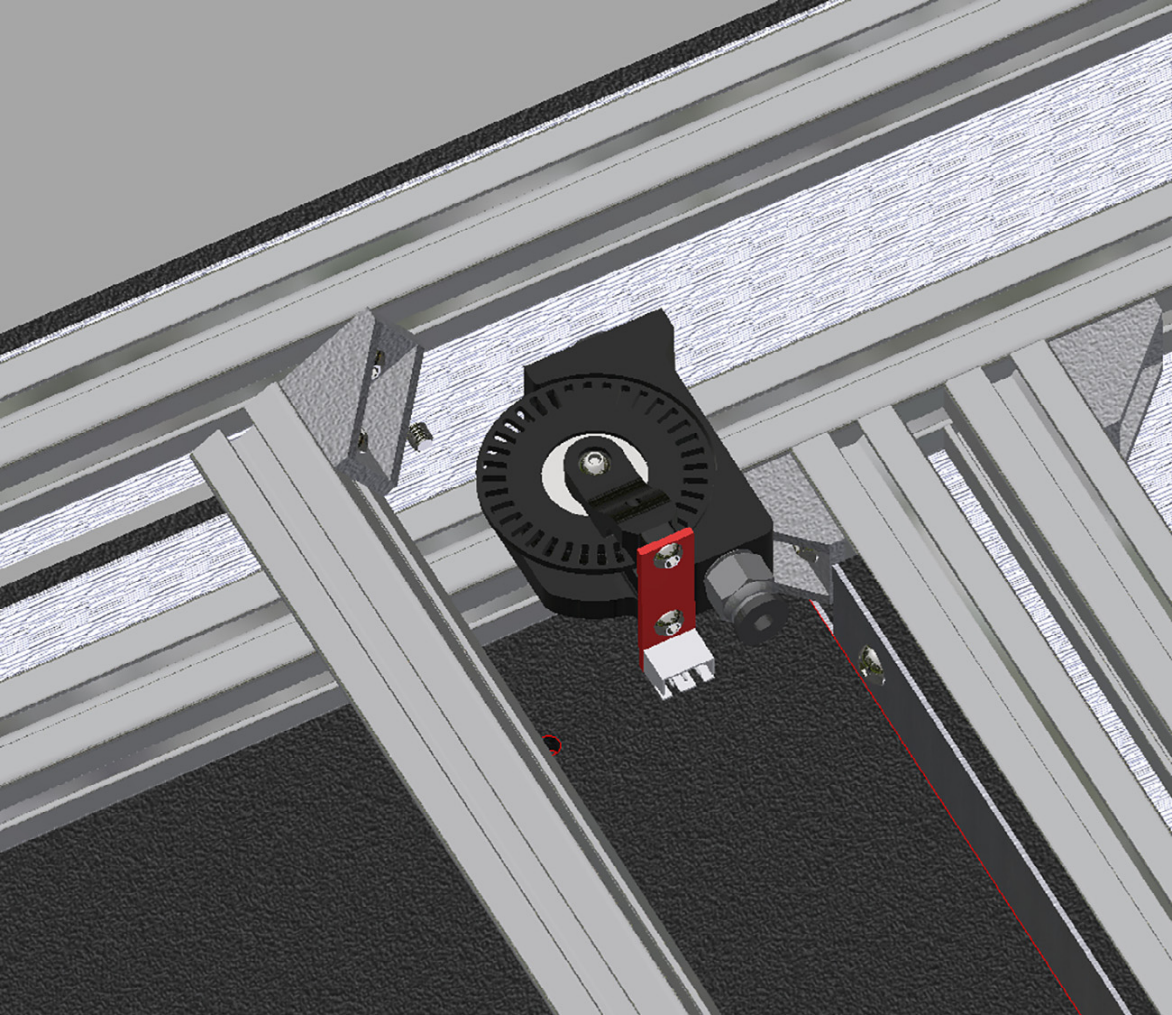
Filament sensor installed on machine.

Since this printer uses materials that print at substantially different temperatures, a jam sensor was added. This is to ensure that the extruder is pushing plastic through the hot end in case the user does not purge the hot end after a high temp print and then attempts to print with a lower temperature material. The sensor assembly is shown in Fig. 23. The sensor uses an optical end stop that counts the number of notches in the wheel that is passing and compares it to what the extruder is supposed to be extruding. Fig. 24 shows the sensor when it is attached to the machine.

**Fig. 25:**
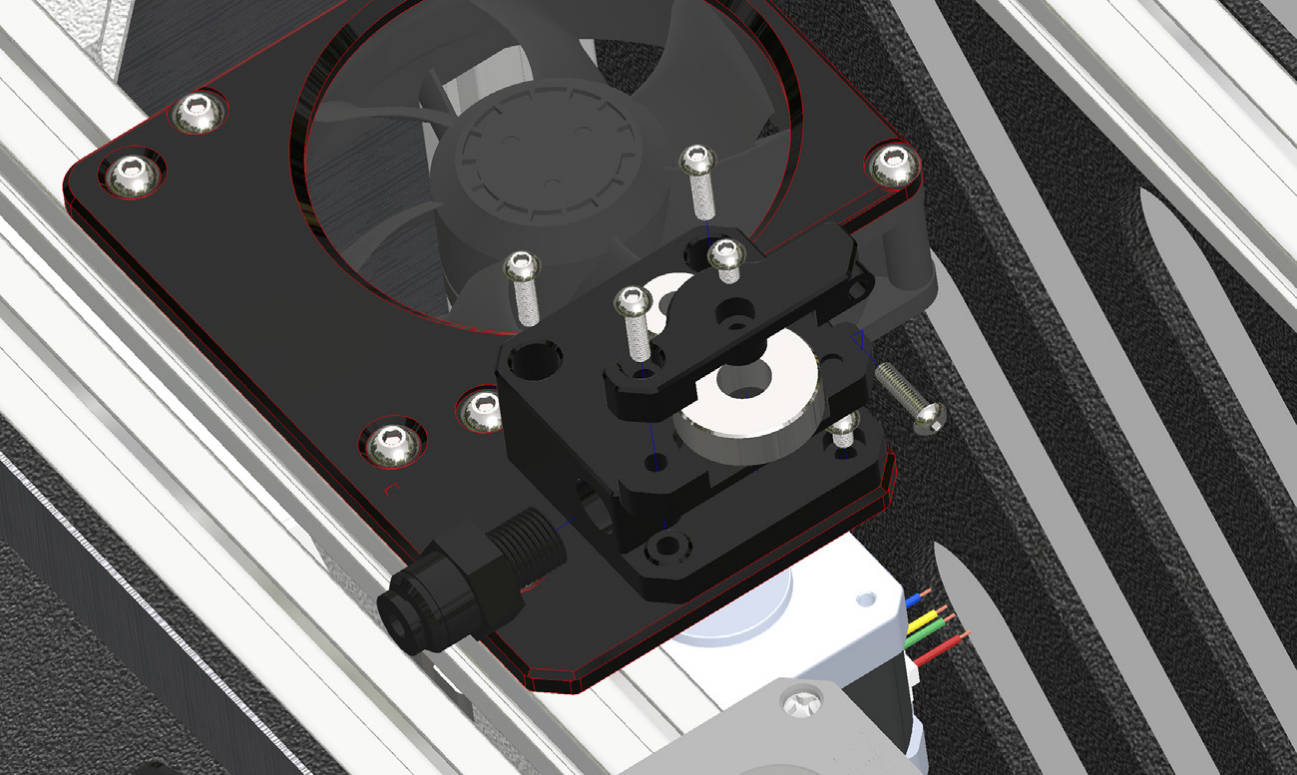
Extruder Assembly.

**Fig. 26:**
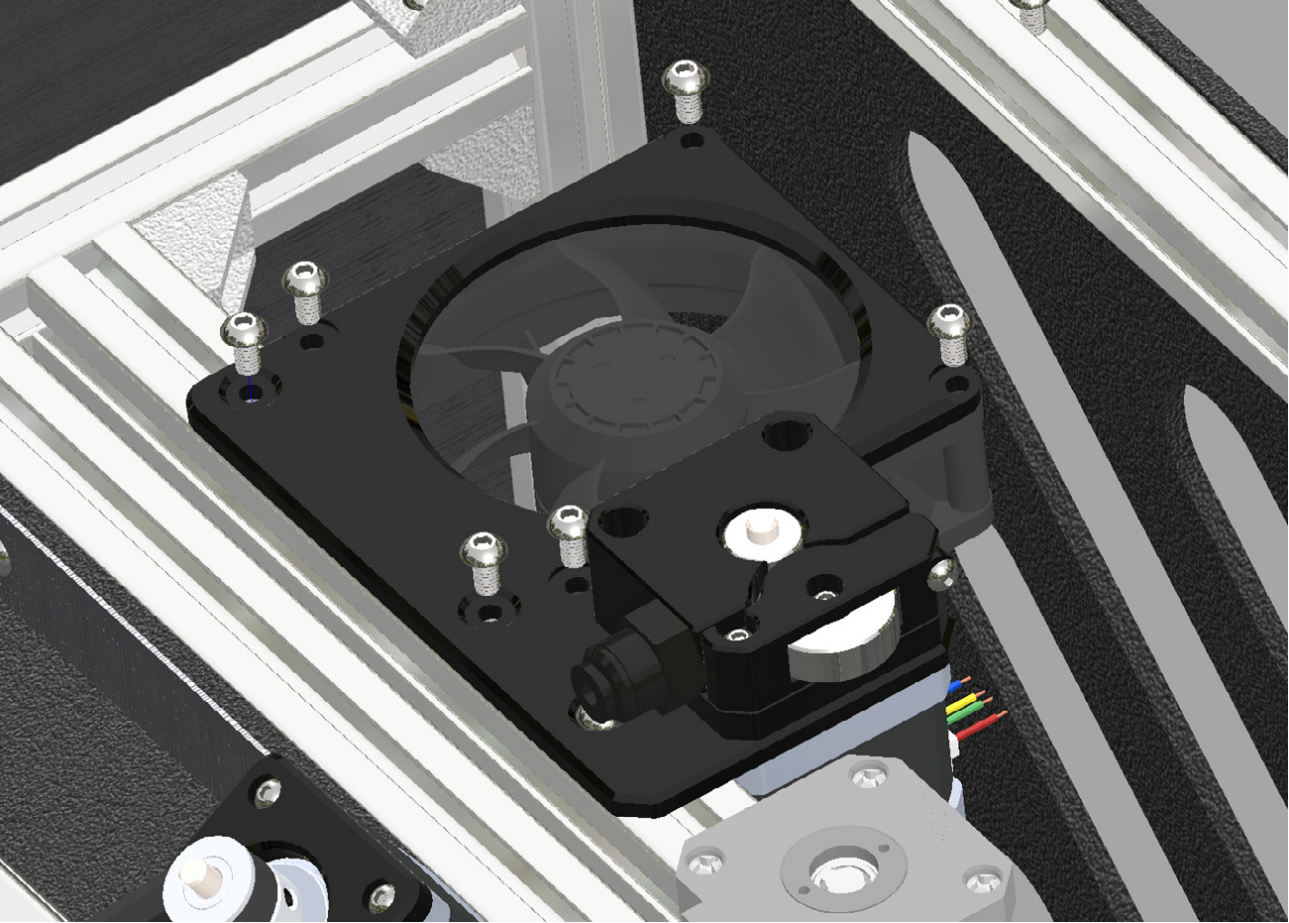
Extruder mounting plate.

Next, the extruder can be mounted to its position near the electronics compartment. The extruder is simple and relies on a simple bolt that can adjust the tension on the filament. A spring can be added along with a longer bolt of the user wants compliance in the system. The mounting is universal as well so other extruders can be used as well. The Bowden coupler that is used is one from E3D and comes with the Bowden V6. The idler is a 608Z bearing or skateboard bearing, and the extruder gear is a 12 mm extruder gear. This assembly is shown in Fig. 25. The mounting plate is shown in Fig. 26.

**Fig. 27:**
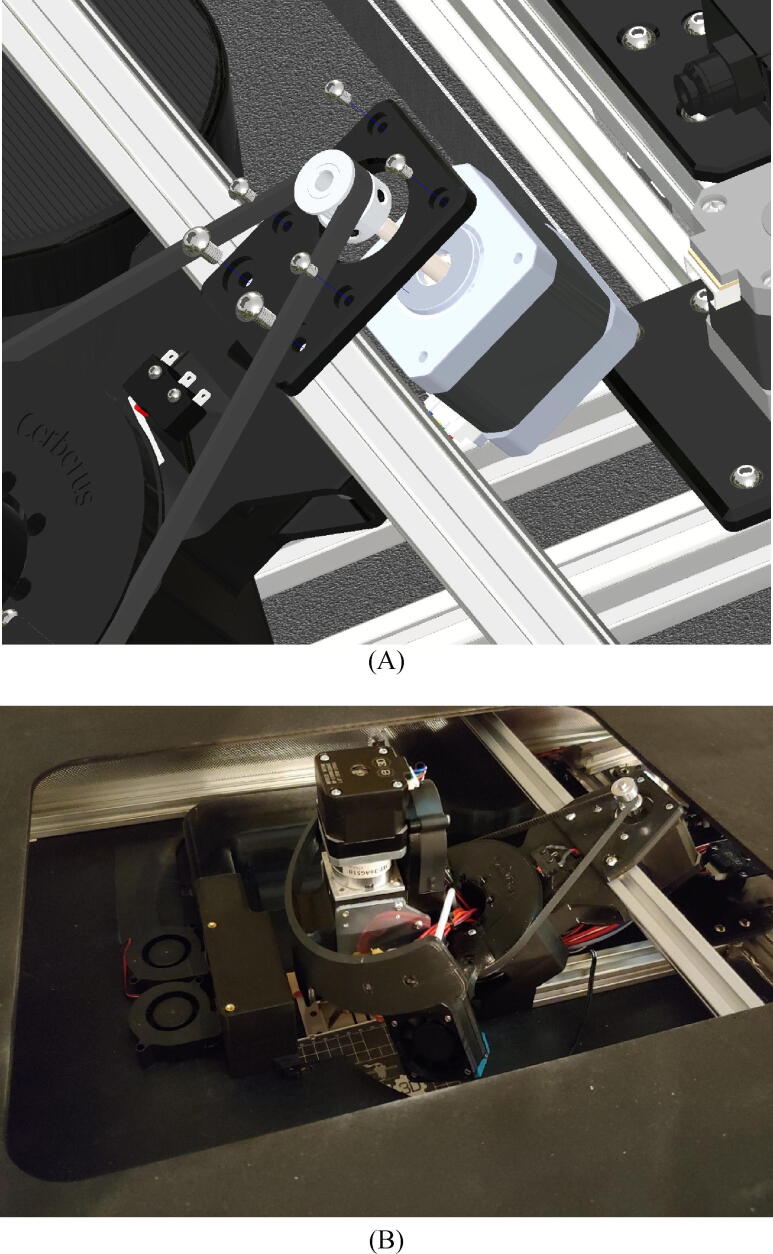
Tool head motor designed (A) and built (B).

To control the rotary tool head, a larger NEMA 17 stepper is used and it is attached as shown in Fig. 27. The GT2 belt is looped through the slots under the center tool head position and the teeth on the belt should engage with itself after it is wrapped around the path given on the pulley.

**Fig. 28:**
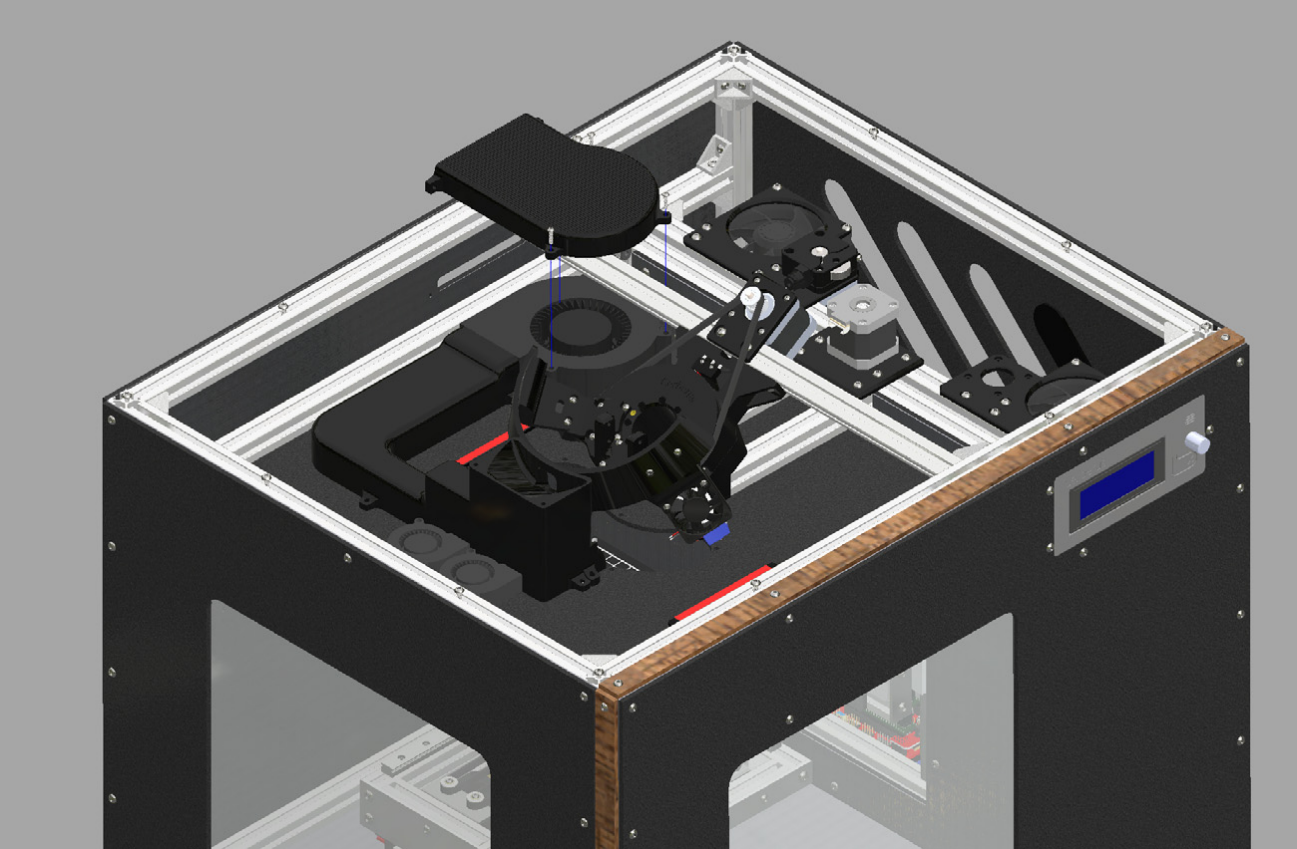
Cooling fans and extra fan.

Extra cooling fans for the center tool head and part cooling fans with corresponding can shrouds are the next set of parts to be installed (Fig. 28). Fan shrouds are attached using bots from below. The small 50 mm radial blower fans are the part cooling fans and the large 120 mm fan is to test better cooling on the hot ends. The large 120 mm fan is not necessary for regular high temperature printing, the 40 mm fan on the V6 is enough.

**Fig. 29:**
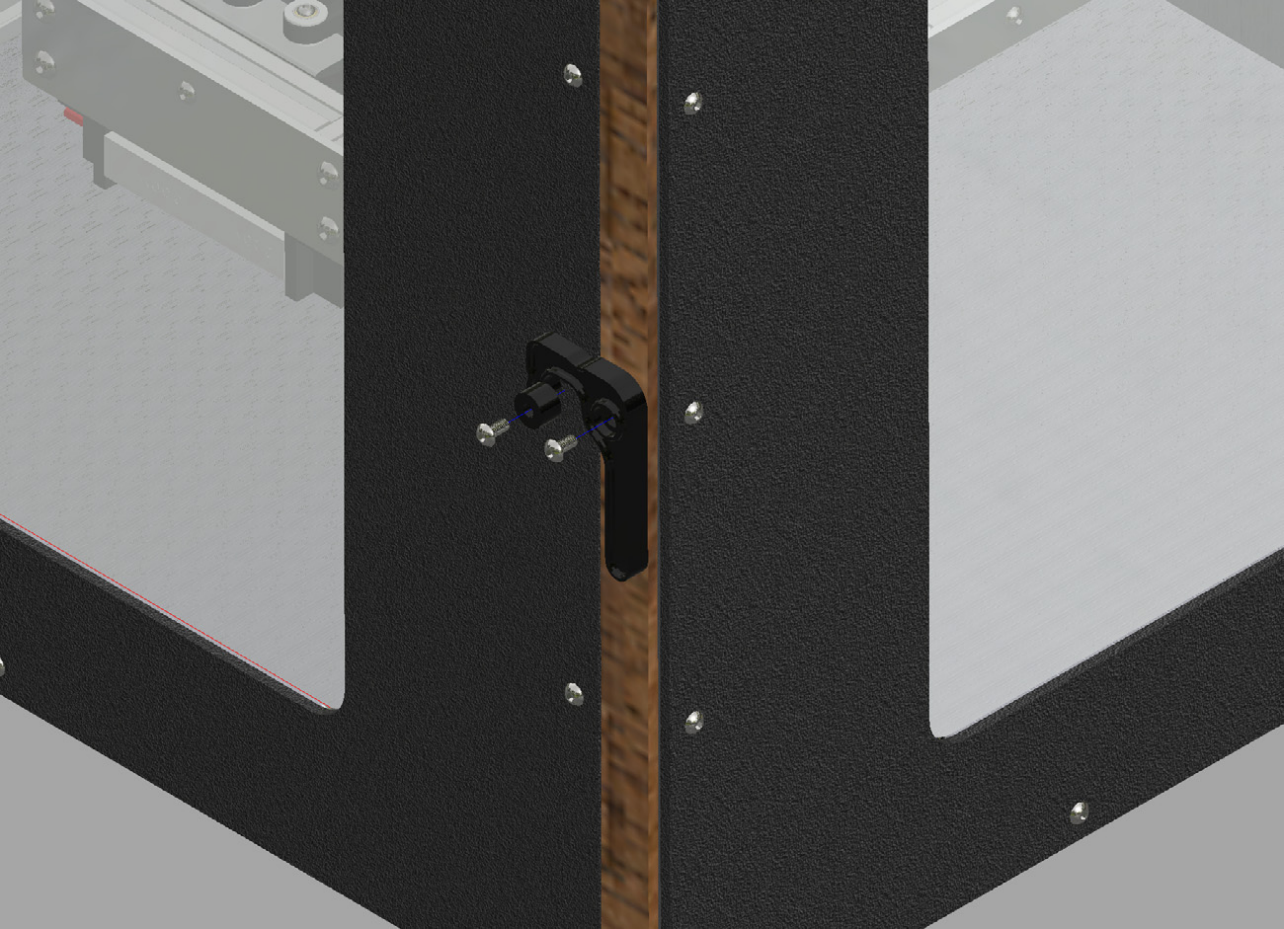
Door latch.

The door latch on this printer is simple and is a compliant bracket that locks around a set pin on the side of the door. Any sort of door latch would work for this. The latch is shown in Fig. 29.

**Fig. 30:**
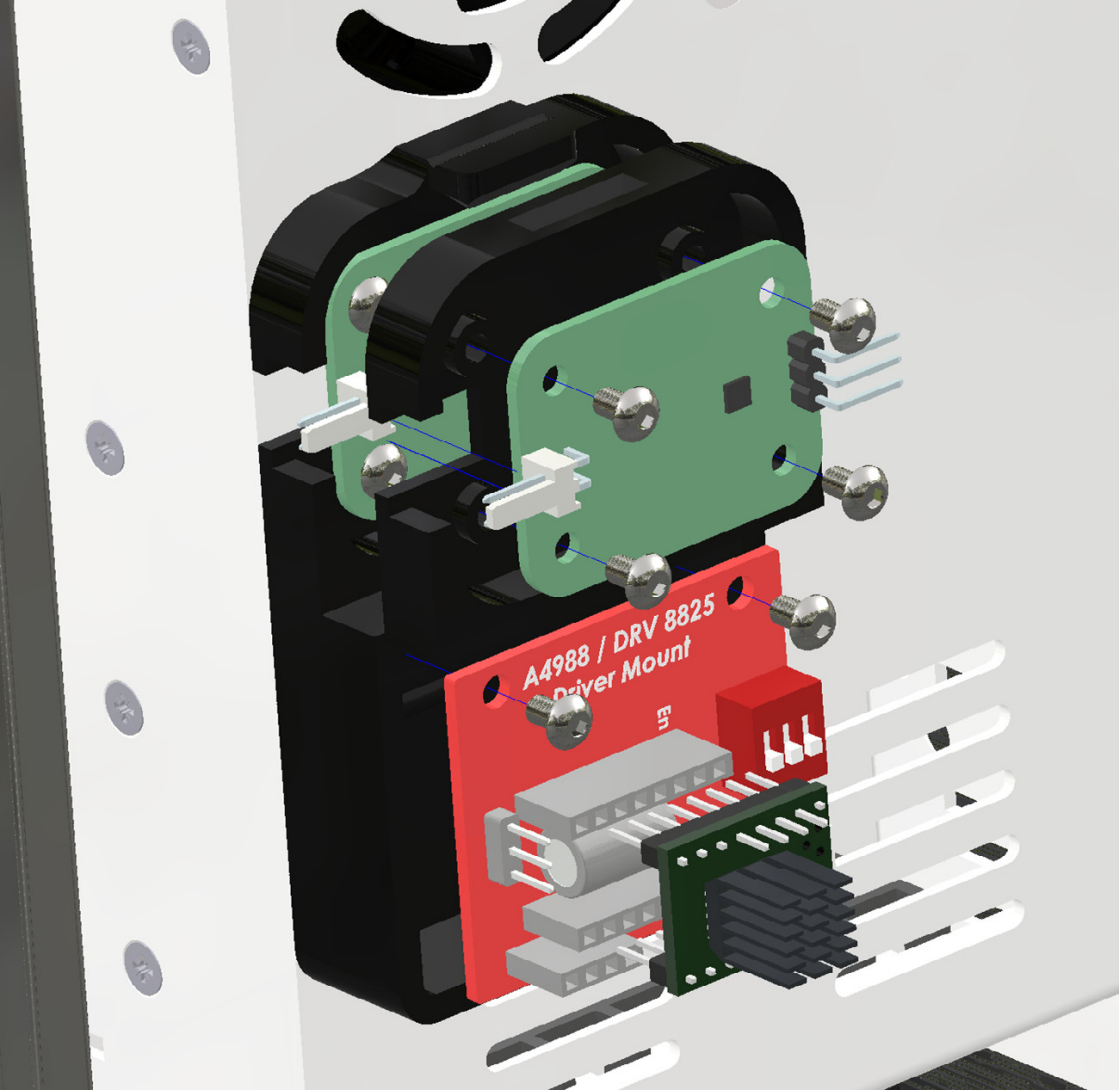
Daughterboard stack.

Since this printer is built to be affordable, it uses an inexpensive RAMPS board. This board is accessible and is a good option to control the printer on a limited budget. If possible, a Duet 3-D controller or others with six stepper drivers would be ideal, but would cost much more. To get around this, two PT100 boards from E3D and one stepper driver board were used to expand the capabilities of the RAMPS board. The assembly for the stack of extra daughterboards is shown in Fig. 30. The PT100 board printed parts snap together and the boards are otherwise bolted together with 4 mm long M3 bolts.

**Fig. 31:**
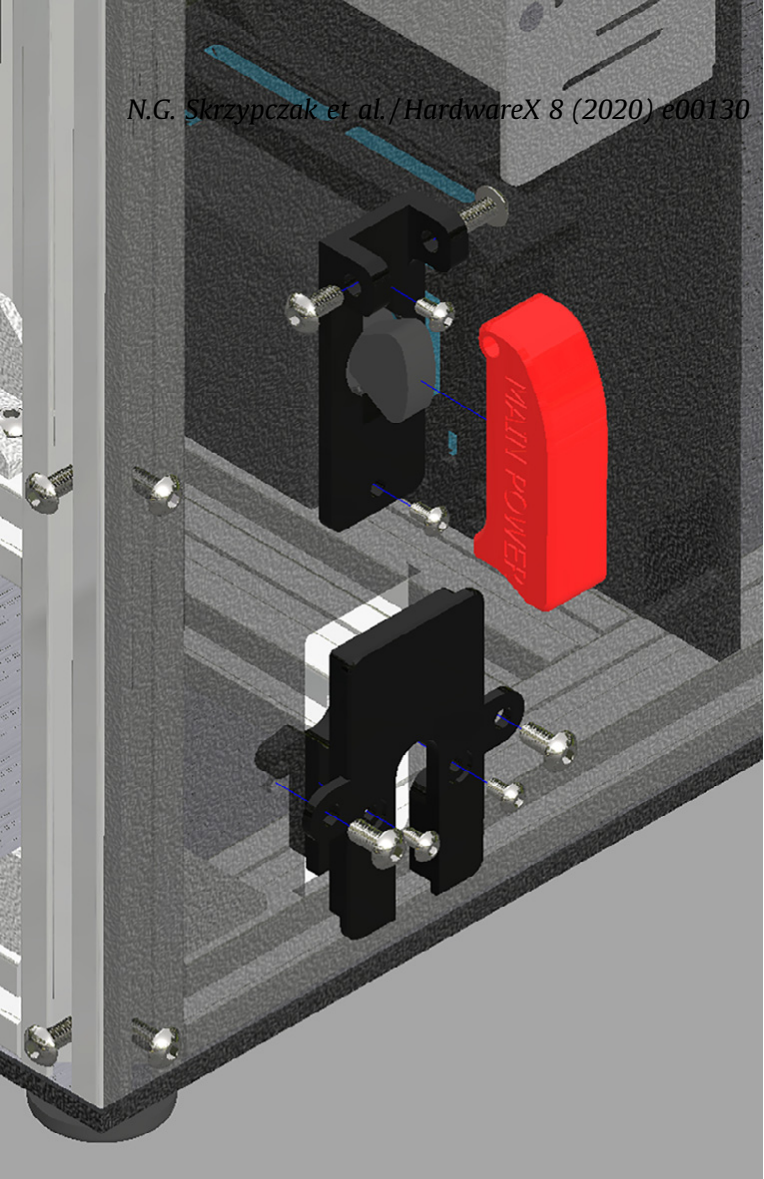
Power delivery parts.

As this 3-D printer has the potential to draw a lot of power, it is necessary to wire the power cable directly into the printer instead of using a small C14 connector with a 5A fuse. To keep the machine safe, a 15A bolt mount breaker is used to control the power. This breaker is used to turn the machine on and off and is protected by a printed switch cover. The main power cable is clamped between two printed parts that keep hands from reaching the mains power behind it. This is shown in Fig. 31.

**Fig. 32:**
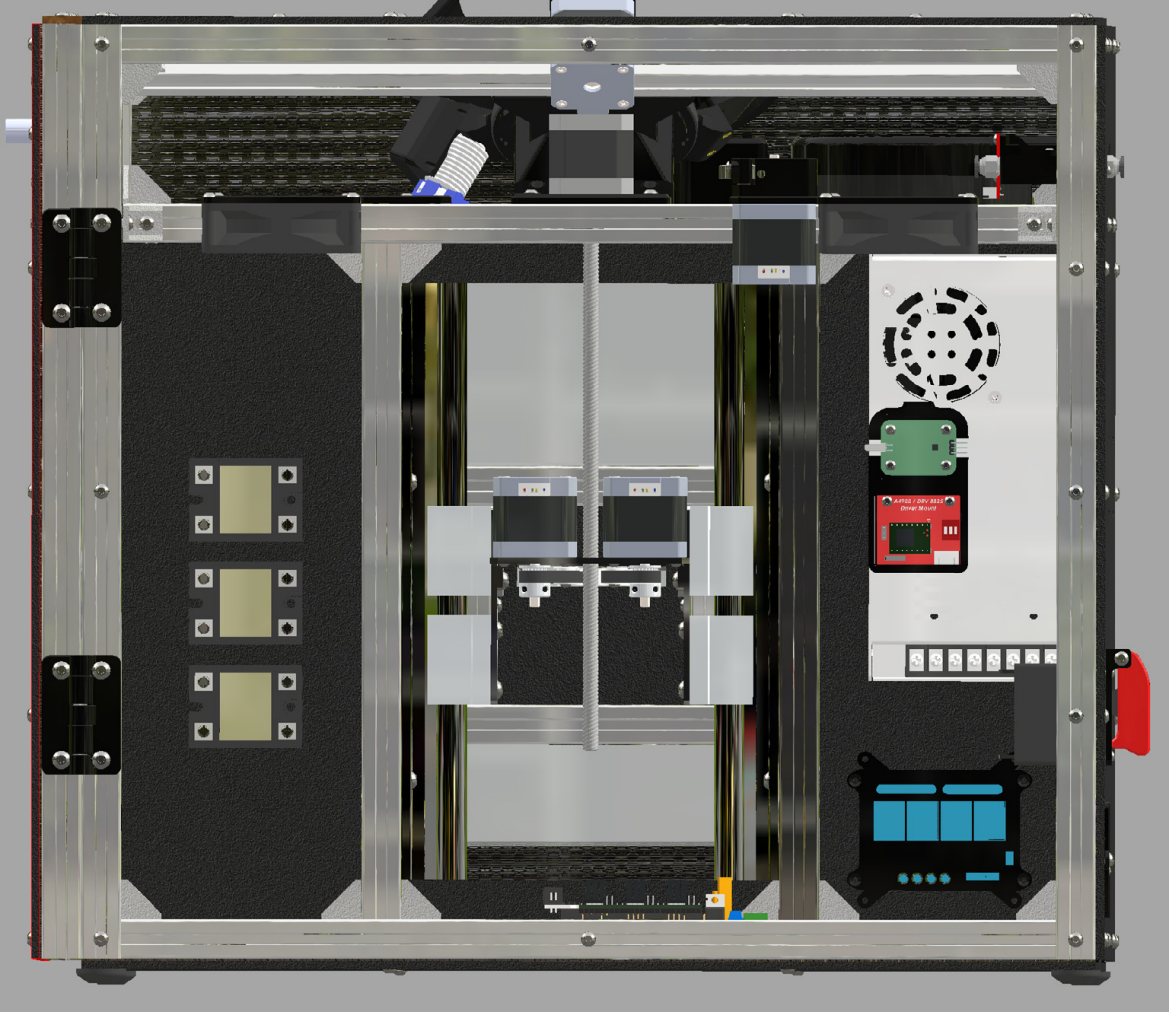
Electronics compartment.

**Fig. 33:**
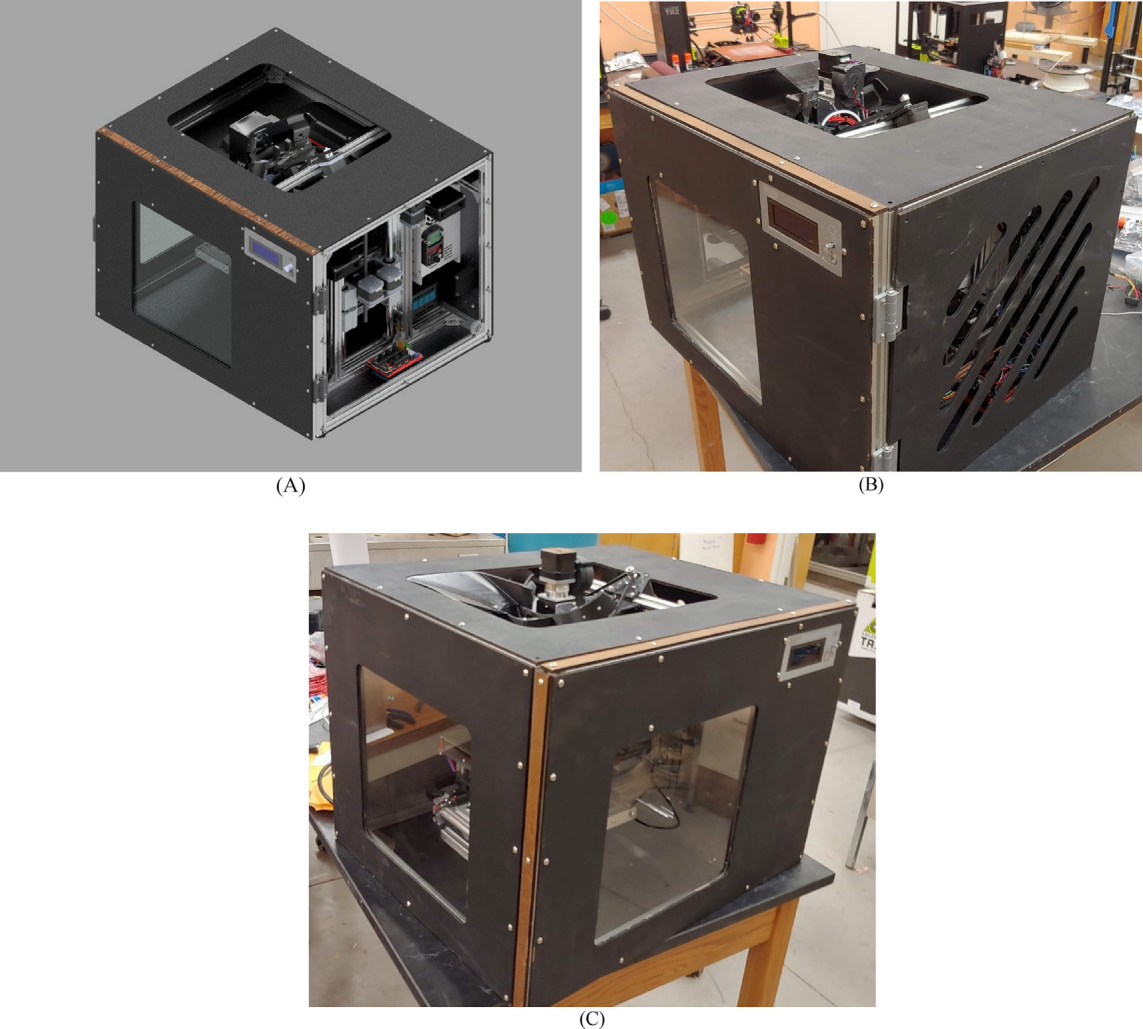
(A) Printer ready for wiring, with front views from (B) right and (C) left.

Before wiring can begin, the various electrical component needs to be mounted. The positions of all the components used is shown in Fig. 32. The exact positions can be changed to fit any needs as long as the center section still allows for the movement of the Z axis up and down. The completed printer assembly minus the rear panel is shown in Fig. 33. The rear panel can be added once the wiring is complete and the machine is tested.

## 5 Electronics

The main electrical components consist of an Arduino Mega 2560 and a RAMPS 1.4 board that runs on a 12v power supply. The more powerful heaters in the system such as the main space heater and the powerful heated bed all run on mains voltage and are controlled using solid state relays. Table 5 explains the wiring for the pins for the RAMPS board.

**Table 5:** RAMPS pins.

RAMPS 1.4 Additional Controls		
Daughterboard	RAMPS 1.4 Pin	Notes
E3D PT100 board primary hot end	5 V	5 V
	GND	GND
	D57	Signal Pin

E3D PT100 board second hot end	5 V	5 V
	GND	GND
	D58	Signal Pin

Turntable Stepper Driver	+12 V	+12 V
	GND (−12 V)	GND
	+5V	+5V
	GND (−5 V)	GND
	D4	Direction
	D5	Step
	D6	Enable

PWM Fan Controllers	Any of the following: D44 D45 D46	Only PWM pins will work to control the fans. +12 V and GND pins are the only fan input.
Optional Relays to control extra fans or	GND	Relay breaks the +12 V
lights	5 V	wire to switch fans/lights
	Any other digital pin available.	on and off.

## 6 Operation instructions

Firmware for the pinout listed in the electronics section of the assembly section is given with the CAD files. The firmware (Repetier-Firmware_Chamber) can be opened in and compiled in Arduino and uploaded to the controller. The rotary tool head must be started in the central position before the printer powers up. The configuration can be uploaded to the Repetier Firmware Configuration Tool to make changes if needed.

Repetier firmware [58] allows for one extra motor controller, and this is used for the rotary tool head. To use it specific start Gcode is used to move it for the bed probing sequence.

Start GCode:
G204 P1 S1; Select motor 1
G28 X; Home X
G28 Y; Home Y
G201 P1 X-58; Move extra motor to the probe position
G28 Z; Home Z
G201 P1 X58; Move extra motor to the furthest tool head position (Center is X0)
G1 Z5 F5000; lift nozzle

Bed adhesion is commonly an issue with printing high temperature materials. This problem is partially counteracted by the heated chamber and high temperature heated bed, but it is not completely eliminated. To help printed parts adhere to the build surface, either nano polymer adhesive from Vision Miner or regular Elmer’s glue stick is used on the glass build surface to keep the printed part stuck to the print surface. An important thing to note about printing high temperature materials is that the parts warp with great force, and if a part is left to sit on the print bed as it cools, it could potentially break the glass. It is important to remove the printed part when the bed is still up to temp. It is also important to remember that the heated parts of this machine, even though they look like regular 3-D printer components, they are much hotter. The heated bed alone when printing polyetherimide (PEI or tradename ULTEM) is almost to the temperature that regular PLA melts at (up to 200 °C) and the hot end is much higher than that (up to a potential 500 °C). The best way to remove the printed parts is to have the machine automatically move the printer bed to a position that it is easy to remove printed parts from it (called “Go to Park Position after Job/Kill). To do this through Repetier host, turn on the setting that tells the machine to go to a park position once a print is done. The park position that is the easiest to remove parts from is X 100, Y 200 and Z180. This puts the bed all the way down and towards the door of the printer, so the part is readily accessible. It is also recommended that when the print is finished it makes a noise or sends a text message to the user, so the bed does not cool down too fast. This can all be done through Repetier host in preferences. Another option is to leave the bed at temp when the print is done as well. Turn off the setting in printer settings that says, “Disable Heated Bed after Job/Kill” and the heated bed will stay on through the host.

Potential hazards that this process presents is the use of both high temperatures and mains voltage. If attempting to build this machine, it is important that the user is confident with working with these dangerous voltages. Make sure the machine is properly grounded and contacts are out of reach or covered. It is also important that the printer is plugged into the wall with a cable that has a ground pin on it and the circuit that the printer is running off of is higher that 15A since the utilization for the circuit in the building should not exceed an 80% utilization. The printer, at full heat up, does not draw a full 15A, but to be on the safe side, a circuit of at least 20A should be used especially if other devices are used on that circuit. The last area of safety to consider is that the printer gets very hot and it is easy to burn hands on various parts of the machine. There is a sensor on the door that helps with moving parts, but the machine stays hot for around 15 to 20 min after use. Proper safety precautions and the use of PPE must be used while using this machine including gloves.

## 7 Validation and characterization

### 7.1 Mechanical testing of high temperature 3-D printed parts

#### 7.1.1 Method

Tensile tests were performed on printed specimens of polyetherketoneketone (PEKK) and PEI/ULTEM materials provided by 3DXTech (Grand Rapids, MI) using the 3-D printing settings shown in Table 6. The specimens were printed according to ASTM 638 type IV standard, which has previously been shown to be adequate for 3-D printing samples [59]. Instron 4206 testing machine was used along with a 300 lb Futek load cell (MODEL LCF455). The extension data was captured by the testing machine based on the crosshead position. Five specimens were tested each for PEKK and PEI/ULTEM samples.

**Table 6:** 3-D printing settings tested on the Cerberus.

Basic Printer Settings				
Material	Nozzle Temperature [°C]	Bed Temperature [°C]	Layer Height [mm]	Part Cooling Fan
PEKK	390	180	0.3	OFF
PEI/ULTEM	380	170	0.3	OFF
Polycarbonate	280	130	0.3	ON or OFF

#### 7.1.2 Results

The PEKK sample had an average peak stress of 77.54 MPa with a standard deviation of 2.75 MPa. The PEI/ULTEM sample had an average peak stress of 80.54 MPa with a standard deviation of 0.81 MPa. The average modulus for the PEKK is 928.78 MPa and for the ULTEM, it is 805.40 MPa. The cross-sectional area for the specimens is 27.1 mm^2^ average with a standard deviation of 0.121 on the width and 0 on the thickness. The peak loads on the PEKK specimens averaged out at 2159.289 N and the PEI/ULTEM averaged out at 2285.478 N.

These properties were closely aligned with values that are expected from these materials. The expected values for the peak strength of PEKK and PEI/ULTEM are 70 N/mm^2^ and 92 N/mm^2^, respectively [60], [61]. Previous work on 3-D printing PEI/ULTEM has shown that strengths were expected to be 46 to 85% of the strengths obtainable by injection molding when printed on a proprietary printer [62]. The results here were slightly better than proprietary printers with more constrained printing parameters, which is similar to results previously observed for acrylonitrile butadiene styrene (ABS) printed with RepRap printers by random makers throughout the world [63]. PEKK is a relatively newer 3-D printing material and material extrusion values appear not to have been published, however, laser sintering-based 3-D printing PEKK provides ultimate tensile strengths ranging from 75 to 90 MPA [64]. The PEKK values were closer to expected than the PEI/ULTEM. This can be caused by variability in the dryness of the material and inconsistencies in layer adhesion. Materials printed on this machine align with others on the market. These values are largely dependent on the material and less on the machine itself if the machine can manage the high temperatures required to print these materials. The values for both PEI and PEKK for ultimate tensile strength, are much higher than what is expected from the commercial filaments available for conventional FFF-based desktop 3-D printers [65], [66], [67]. The PEI and PEKK even have tensile strengths substantially higher than polycarbonate (PC), which is generally the strongest material available for FFF and fused particle fabrication (FPF)/fused granular fabrication (FGF)-based standard printers [65], [68], [69].

### 7.2 Thermal testing of COVID-19 Maker mask

**Fig. 34:**
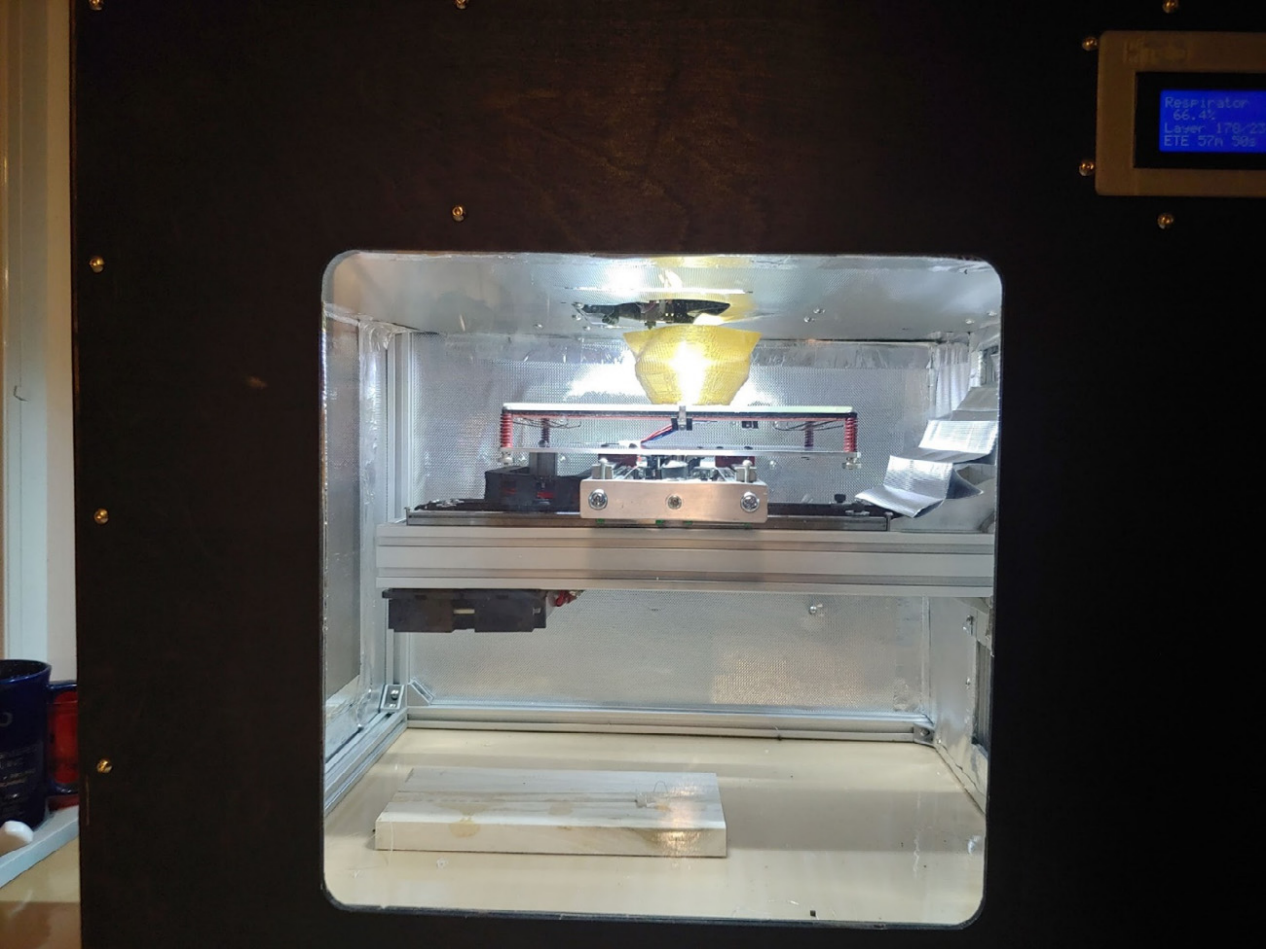
PEKK High Temperature Printed Mask.

**Fig. 35:**
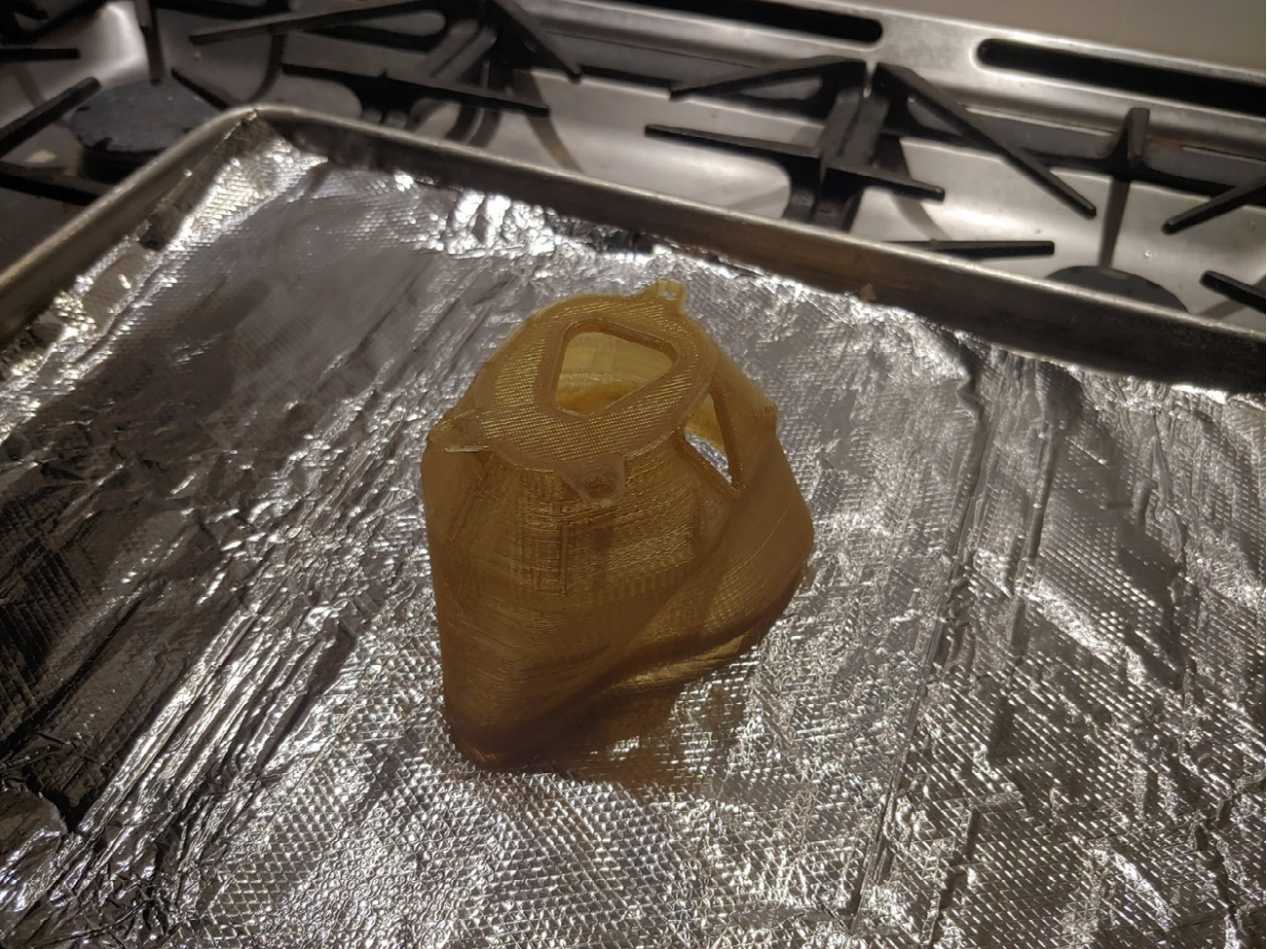
Sterilization Test Results.

To demonstrate a potential COVID-19 use case for this machine, a reusable face mask was printed on the machine out of PEKK as shown in Fig. 34. The mask was then put into an oven at 120 °C for 30 min. The results of the test are shown in Fig. 35.

The test was successful and showed no observable deformation of the thin walled mask from Maker Mask [70]. There is potential for PEKK to be annealed as well, and according to the 3DXTech filament specification sheet, after the annealing process the maximum operational temperature for the part is 260 °C. This operating temperature is 110 °C higher than that of the non-annealed part. The capabilities for this material are already well known but methods for printing with this material were what this test was aimed at accomplishing.

## 8 Machine capabilities, future work and conclusions

The Cerberus showed promising results for 3-D printing PEI/ULTEM and PEKK. The heated chamber, high temperature components, and isolated electronics all allowed the machine to print these materials and others similar should perform similarly.

Capabilities of the Cerberus open source high-temperature 3-D printer are:

1. High temperature 3-D printing (up to 500 °C nozzle temperature, 200 °C bed temperature)
2. Scalable tool heads where weight and size does not affect print quality
3. External electronics to save costs on cooling components and improve reliability and functionality
4. Potential for dual extrusion of high temperature materials or soluble supports for high temperature thermoplastics.
5. Auto bed leveling and manual bed leveling through probe integration
6. Freedom to add more functionality to the tool heads.

Drawbacks of the design:

1. Tools are difficult to remove or change currently.
2. A shorter extrusion path is needed to print some materials such as carbon fiber filled materials.
3. Dual drive extruder gears will help in printing with carbon fiber materials.
4. Uses FFF instead of FPF/FGF, the latter of which can make use of far less-costly feedstocks such as pellets [71], [72], [73], [74], [75], [76] and is more easily adapted for recycled 3-D printing [77], [78], [79], [80], [81].

These drawbacks can be overcome in future revisions of this open source 3-D printer. Design files for a test quick release tool system is also included in the design files posted along with a direct Bowden combination drive system for the V6 hot end. Additional functionalities for this Cerberus printer can be a pellet extruder, an automatic nozzle cleaner, and a method to print continuous fibers at high temperatures. In addition, a pellet extruder will allow for recyclability of either high temperature plastics or others like PETG or PLA plastic. A nozzle cleaner will allow the printer to clean the nozzle before the print will start. The high print temperatures cause the nozzle to ooze plastic when the print head is not in use and can sometimes peel the first layer off the print bed when a print begins. The last future functionality that will expand the capabilities of the machine is continuous carbon fiber. In combination with high temperature thermoplastics, continuous fiber can greatly improve the strength of the printed parts.

As compared to other printers on the market, the Cerberus is much more affordable than all high-temperature printers, and yet it can print materials that are difficult (or impossible) to print on a typical low-temperature desktop 3-D printer. Thus, the Cerberus offers the potential for scalable distributed manufacturing. The high temperature capability enables it to print thermally sterilizable products such as the face mask demonstrated here for pandemic PPE. In addition, the high strengths capable for the PEI and PEKK materials also lend themselves to a long list of engineering applications and products that are not viable on conventional FFF-based desktop 3-D printers.

## Declaration of Competing Interest

The authors declare that they have no known competing financial interests or personal relationships that could have appeared to influence the work reported in this paper.
